# Decoding the role of mesothelin in tumor dynamics and targeted treatment innovations

**DOI:** 10.1186/s43556-025-00379-z

**Published:** 2025-12-03

**Authors:** Roberto Silvestri, Emanuela Colucci, Margherita Piccardi, Stefano Landi, Federica Gemignani

**Affiliations:** Department of Biology, University of Pisa, Pisa, Italy

**Keywords:** Mesothelin, Targeted therapy, Tumor microenvironment, CAR-T cells, Bispecific antibodies

## Abstract

Mesothelin (MSLN) is among the most studied cancer-related antigens, and it is extensively studied as a therapeutic target for the treatment of various malignancies, including pleural mesothelioma, pancreatic ductal adenocarcinoma, and ovarian cancer. However, despite the development of many MSLN-targeting strategies, such as antibody–drug conjugates (ADC), bispecific antibodies, and CAR-T cells, clinical responses have remained limited, underscoring the need for a deeper understanding of MSLN biology. Over the past decades, many studies have highlighted a link between MSLN and cancer progression and its association with specific features within the tumor microenvironment (TME). More recently, mechanistic evidence has emerged showing the involvement of MSLN in the establishment of key malignant features, such as the epithelial-to-mesenchymal transition (EMT) and matrix metalloproteinase 7-mediated remodeling of the extracellular matrix (ECM). Furthermore, these studies also show a direct role for MSLN in the immunosuppressive polarization of the TME through the interaction with CD206 macrophage receptors (leading to an M2-like polarization) and by promoting the transition of mesothelial cells into specific cancer-associated fibroblasts (CAFs). This review synthesizes current evidence on MSLN transcriptional regulation and its functional implications in invasion, metastasis, and immune evasion. We also summarize ongoing therapeutic strategies targeting MSLN and discuss how TME-driven resistance mechanisms are shaping the next generation of MSLN-directed therapies. By integrating molecular insights with translational perspectives, this work provides a comprehensive overview of MSLN biology and its emerging therapeutic relevance in cancer.

## Introduction

Mesothelin (MSLN) is a 40 kDa protein bound to the cell surface through a glycophosphatidylinositol (GPI) anchor [1]. Under physiological conditions, MSLN expression is low and restricted to mesothelial-derived tissues, such as the pleura, pericardium, and peritoneum, where its biological role remains largely unknown. The membrane-bound form may undergo further proteolytic processing by a range of proteases, including members of the ADAM, MMP, and BASE families, resulting in the release of a soluble form of MSLN (also known as “soluble mesothelin-related peptide”, SMRP) [2].

Over the years, high levels of MSLN expression have been reported in tissues and peripheral blood across a wide range of malignancies, including mesothelioma, ovarian, pancreatic, and esophageal cancers [3]. Furthermore, several studies have linked MSLN to key tumorigenic processes. Indeed, MSLN expression has been associated with the epithelial-to-mesenchymal transition (EMT), cellular proliferation, survival, and cell motility [4]. These characteristics have made MSLN a promising diagnostic and prognostic biomarker for malignancies that overexpress MSLN. Specifically, in pleural mesothelioma (PM), the accuracy of MSLN as a biomarker has been evaluated across different matrices (mostly serum, plasma, and pleural fluids) and among different groups of individuals (mesothelioma patients, individuals affected by benign respiratory diseases, and healthy subjects). In most studies, MSLN showed high specificity (ranging from 81 to 97%) but moderate sensitivity (58–66%) [5]. Confounding factors may play a critical role in this context, including well-known ones such as age, body mass index, and renal clearance, as well as others that remain to be elucidated [6].

For the same reasons outlined above, MSLN has also emerged as one of the most explored therapeutic targets in a wide range of cancer types [7]. In this respect, the first targeting attempt that progressed to clinical trials was carried out by Hassan et al. in 2002 using an anti-MSLN recombinant immunotoxin [8, 9]. These trials showed that the drug was generally well tolerated, but its clinical efficacy as a monotherapy was limited, with few partial responses and mostly disease stabilization. Since then, many targeting strategies have been developed and tested both in vivo and in clinical trials. Among the most promising approaches are those based on CAR-T cells, in which patient-derived T lymphocytes are engineered to express chimeric antigen receptors recognizing MSLN and, in some cases, to target multiple antigens within the tumor microenvironment (TME) [10, 11]. However, even CAR-T cell therapies face many obstacles, including off-tumor toxicity, limited trafficking, and immunosuppressive TME, that limit their clinical efficacy [12], highlighting the need for a better understanding of MSLN functional roles in tumor progression and TME remodeling.

In this review, we present an integrated overview of MSLN biology, from its transcriptional regulation across malignancies to the mechanisms by which it promotes metastatic dissemination, including cell-to-cell adhesion, extracellular matrix (ECM) degradation, and EMT. We further highlight MSLN’s role in shaping the TME by analyzing its association with specific microenvironmental features and the most up-to-date evidence on its mechanistic contribution to the establishment of an immunosuppressive *milieu*. Finally, we examine the landscape of current MSLN-targeted therapies, with a special emphasis on emerging cell-based immunotherapeutic approaches and discuss future directions for optimizing MSLN-targeted interventions.

## The molecular biology and expression regulation of mesothelin

MSLN was discovered in 1992 by Chang and collaborators as an antigen associated with ovarian cancer and other malignancies [1, 13]. This antigen, originally named CAK1, was identified as a 40 kDa protein bound to the cell surface via a GPI anchor [1]. Aside from its cancer-associated expression, MSLN is differentially expressed in multiple tissues. It has been detected in mesothelial-derived tissues such as the pleura, pericardium, and peritoneum [3]. Multiple studies, using integrated omics approaches, have reported high expression of MSLN in ciliated cells of the bronchus and fallopian tube, in microvilli of the placenta, and in squamous epithelial cells of the tonsil. Medium expression has been observed in ciliated cells of the nasopharynx, as well as in endocrine cells and mucosal lymphoid cells of the colon, rectum, and endometrium. Low expression has been detected in squamous epithelial cells of oral mucosa and cervix, as well as keratinocytes and melanocytes [14, 15] [Human Protein Atlas proteinatlas.org].

In 1996, the cDNA encoding this protein was cloned and expressed [16], and its promoter was characterized. In this context, studies conducted mostly on cancer cells revealed the presence of two essential motifs, an SP1-like and an MCAT, that drive gene expression and may contribute to the tissue-specificity observed for MSLN. The same study also suggested that the transcription factors YAP1 and KLF6 may modulate MSLN expression [17].

**Fig. 1:**
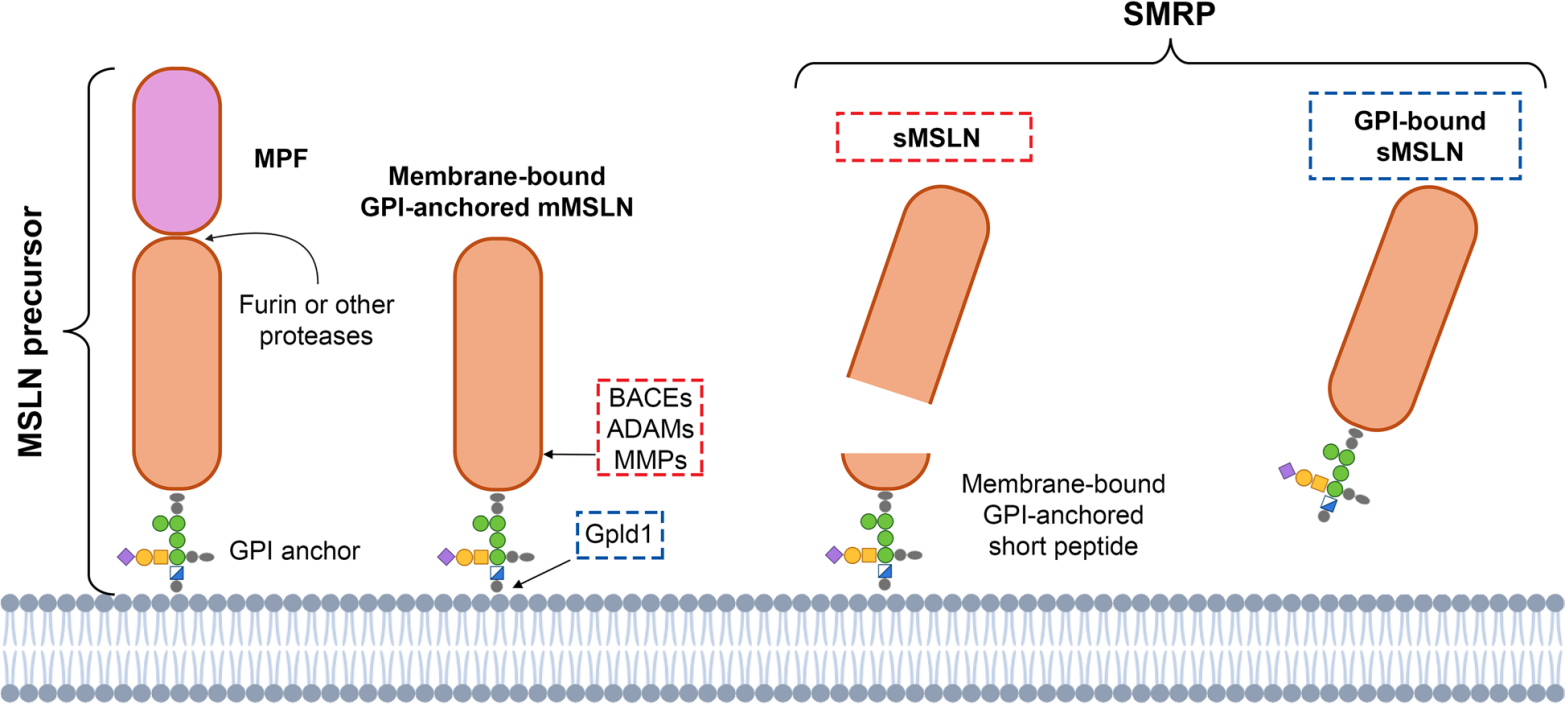
Schematic representation of MSLN isoforms. MSLN is shown in all its known isoforms. On the left, the 71-kDa precursor form is depicted, including the GPI anchor, the 40-kDa MSLN, and MPF. The magenta oval at the top represents MPF, which is released following cleavage by furin or other proteases. The mature mesothelin (mMSLN) exists in multiple isoforms: a membrane-bound form and SMRP, the latter including GPI-anchored and non-anchored soluble variants (sMSLNs). Specifically, the proteases BACEs, ADAMs, and MMPs generate the short GPI-anchored peptide and sMSLN, whereas Phospholipase D Gpld1 cleaves the GPI anchor from the membrane, releasing the GPI-bound soluble form

The *MSLN* gene encodes a 71 kDa GPI-anchored precursor. This precursor is cleaved by furin to produce the 30 kDa Megakaryocyte Potentiating Factor (MPF) and the mature 40 kDa MSLN [18] (Fig. 1). Joseph et al. further analyzed MSLN post-translational processing, highlighting that, in the absence of furin, other proteases can mimic its activity, releasing MPF, and converting the precursor protein into the mature MSLN [19]. Other cleavage events may occur at the C-terminus of MSLN, mediated by different families of proteases (BACEs, ADAMs, and MMPs), which are particularly expressed in malignant contexts [2]. The activity of these proteases leads to the release of soluble MSLN, also known as soluble mesothelin-related peptide (SMRP), with a short GPI-anchored peptide remaining attached to the membrane after cleavage.

Recently, a study by Dangaj et al. has also shown that phospholipase D can cleave the MSLN GPI anchor, releasing a soluble form of MSLN still bound to the GPI. Interestingly, the mannose component of the GPI anchor can bind to the CD206 receptor on macrophages, suggesting a potential immunomodulatory activity for MSLN [20].

**Fig. 2:**
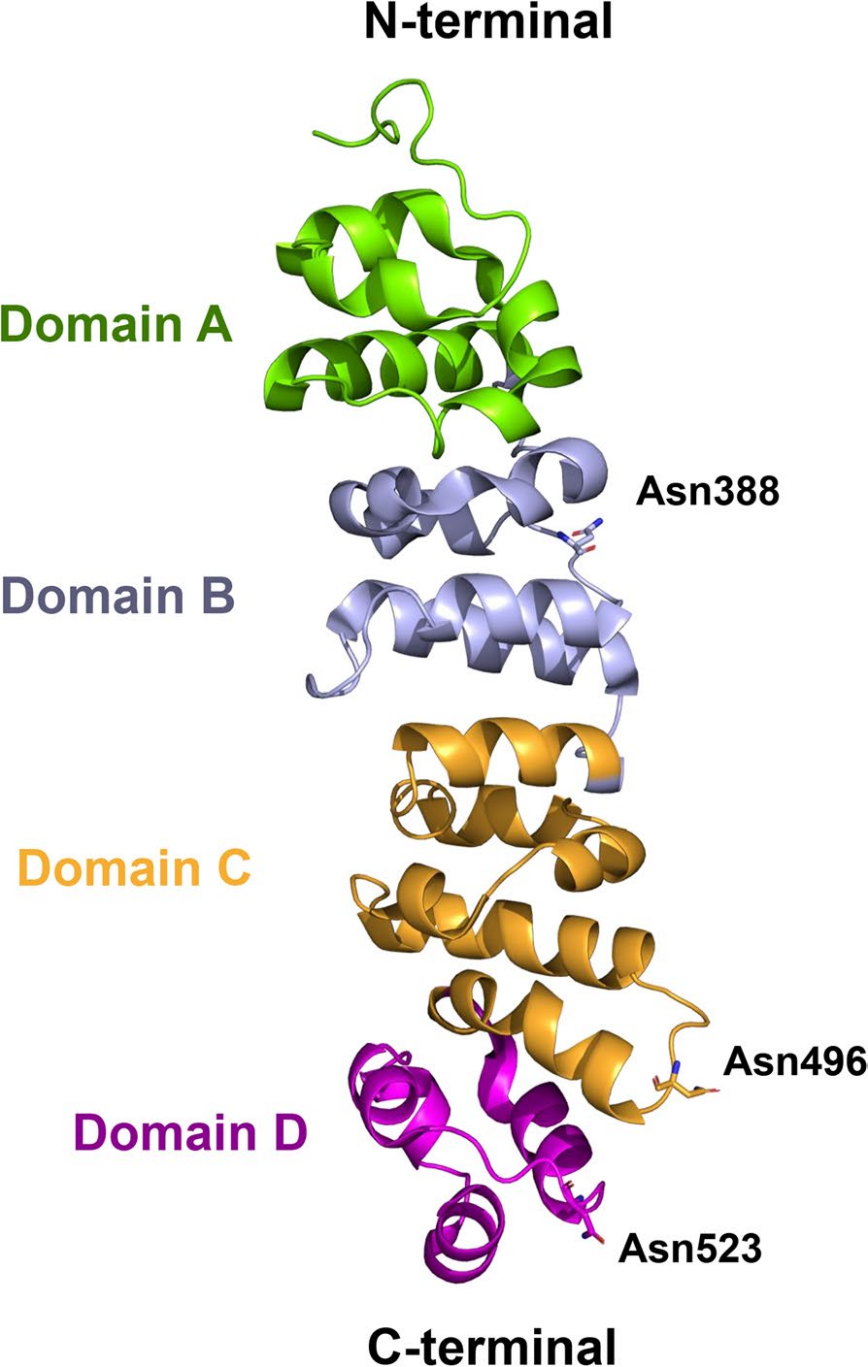
3D structure of MSLN. Cartoon representation of the crystal structure of MSLN (PDB ID: 8XC3), showing its organization into four domains: the N-terminal Domain A (green), Domain B (light purple), Domain C (orange), and Domain D (magenta), which contains the C-terminus. The three asparagine residues involved in N-glycosylation are depicted in licorice representation

The first crystal structure of MSLN was reported in 2012, when only the short epitope involved in CA125 binding was resolved in complex with the anti-MSLN antibody MORAb-109, which prevents this interaction [21]. It took eleven more years to obtain the full-length structure of MSLN through X-ray crystallography [22]. This study revealed that MSLN is composed of 17 short α-helices arranged into four α-helical domains, forming a compact, right-handed, elongated spiral (Fig. 2). The protein is heavily N-glycosylated at three conserved sites. The GPI anchor at the C-terminus enables lateral mobility within the membrane. The surface-exposed regions include positively charged epitopes that bind CA125 and are recognized by therapeutic antibodies. The MSLN ectodomain (residues ~ 296–580) contains four repeating α-helical motifs, each comprising ~ 60–70 amino acids. These domains are arranged linearly to form a right-handed superhelix. A DALI structural homology search using the crystal structure of human MSLN (PDB ID: 8CX3) revealed no close structural homologs, suggesting that the α-helical solenoid architecture of MSLN is unique or rare among human proteins.

The four-domain organization provides an extended surface ideal for protein–protein interactions. Domains A and B form the high-affinity interface with CA125, while domains C and D, located closer to the membrane, are involved in structural stability, glycosylation, GPI-anchor interactions, and localization of the sequence recognized by proteases.

While the crystal structure did not directly determine the composition of the glycans, their identity was inferred from glycans isolated from cell culture supernatants and patient-derived ascitic fluid. The predicted N-linked glycosylation sites—based on N-X-S/T motifs—were confirmed at Asn388, Asn496, and Asn523 [16, 23].

Despite rapid progress in defining the structure and post-translational processing of MSLN, its physiological role remains largely elusive. Indeed, knockout (KO) mice showed that lack of MSLN did not lead to any vital or reproductive abnormalities [24], ruling out a crucial role for this protein under physiological conditions. Some authors have suggested that MSLN could play a role in cell adhesion and the trafficking of molecules into and out of the peritoneal cavities [16]. A more recent study conducted on the mouse homologous gene (named Expressed in Renal Cancer, *ERC*) suggested a structural role for MSLN in epicardial and pleural development at key stages of heart and lung formation [25]. In wild-type (WT) mice, ERC expression in the epicardium preceded pleural expression by six days. Structural analyses showed that ERC-KO mice had shorter and less dense microvilli than WT, although functional tests of cardiac and pulmonary function revealed no significant physiological differences [25]. Overall, these findings suggest that MSLN may exert adhesive or structural functions in a context-dependent manner, but further studies are needed to fully elucidate its biological role.

## Mesothelin in tumor pathology and dynamics

While our understanding of the physiological functions of MSLN remains limited, there is considerable interest in defining its role in tumor pathology. MSLN is markedly overexpressed in several malignancies, including pancreatic ductal adenocarcinoma (PDAC), ovarian cancer, non-small cell lung cancer (NSCLC), pleural mesothelioma (PM), and others. This selective overexpression in tumors, along with its restricted distribution in normal tissues, makes MSLN both a potential biomarker for cancer detection and prognosis and a promising target for the development of MSLN-directed therapies. Building on the discussion of its physiological functions, the following section explores how MSLN contributes to tumor biology examining its expression patterns across different cancer types, its functional roles in tumorigenesis, and its involvement in metastatic dissemination and remodeling of the tumor microenvironment. Together, these insights provide a framework for understanding how MSLN may influence not only cancer progression but also the emergence of therapeutic resistance in MSLN-expressing tumors.

### Expression patterns of mesothelin in cancer

Studies on MSLN in PDAC have been extensively used to explore how MSLN expression is regulated, providing insights into its underlying mechanisms and shedding light on potential pathways that could be targeted for therapeutic intervention.

**Fig. 3:**
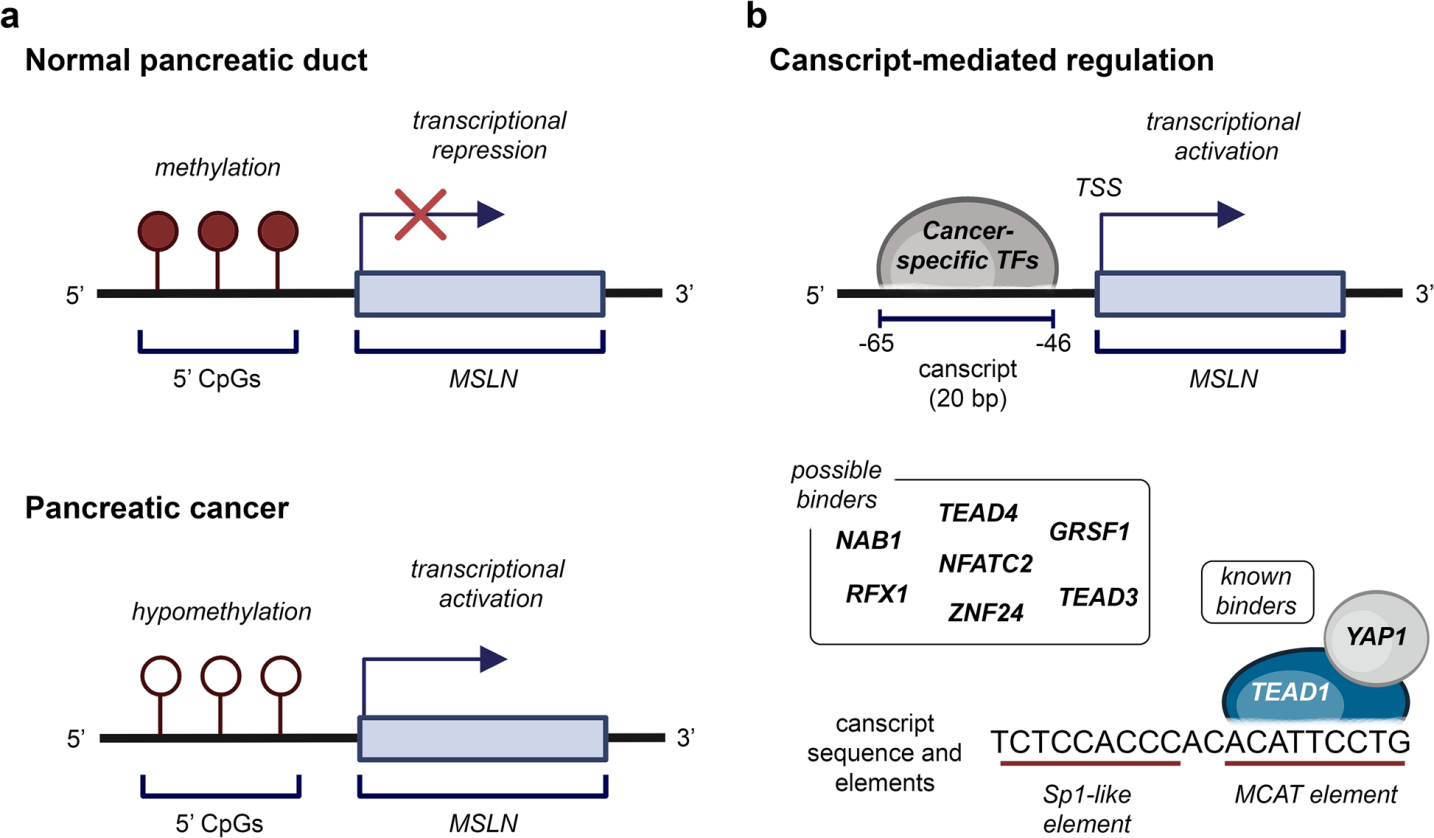
Mechanisms of *MSLN* transcriptional regulation. In normal pancreatic duct, the methylation of the 5’ CpGs limits the expression of *MSLN*. Hypomethylation of this region has been observed in pancreatic cancer, leading to transcriptional activation (a). A promoter-like sequence, Canscript, located upstream of the transcription start site (TSS) and composed of an Sp-1 like and an MCAT element, functions as a binding site for cancer-specific transcription factors and is in part responsible for the *MSLN* overexpression in malignant tissues

In the study by Sato and colleagues (2003), *MSLN* is one of the genes overexpressed in pancreatic cancer due to selective DNA hypomethylation. While *MSLN* is normally methylated and transcriptionally silent in healthy pancreatic tissue, its activation in PDAC suggests a pivotal role for epigenetic deregulation in its expression [26] (Fig. 3a). A crucial regulatory element in the context of malignant *MSLN* expression is an 18-bp sequence located upstream of the transcriptional starting site (TSS), named CanScript. Originally identified in pancreatic cancer, CanScript has been reported to play a key role in other malignancies as well, such as mesothelioma and ovarian cancer, where it strongly enhances *MSLN* expression [27] (Fig. 3b). Further studies conducted in PDAC and ovarian carcinoma have identified CanScript-like sequences upstream of several overexpressed genes, including *FXYD3*, *MUC1*, and *TIMP1,* highlighting its importance in cancer development [28]. Structurally, CanScript is comprised of SP1-like and MCAT elements that seem to be crucial for the *MSLN* overexpression [27]. Indeed, key regulators of gene expression, known to participate in the development of a malignant phenotype, have been reported to bind to the MCAT element. Among these are the TEA Domain Transcription Factors (TEADs), in particular the TEAD1/YAP1 complex, as well as other transcription factor families such as Myc and AP-1 [29]. Despite this evidence, however, the exact mechanisms orchestrating the *MSLN* expression are not yet fully understood. Additional data come from studies on PDAC, which show that NF-κB, activated by MSLN, can also act as a transcriptional activator of *MSLN* itself, establishing a positive feedback loop that promotes tumor progression by conferring resistance to tumor necrosis factor alpha (TNF-α)–induced apoptosis [30].

MSLN expression is also regulated at a post-transcriptional level, with miR-198 playing a key role in suppressing MSLN expression and inhibiting the MSLN/NF-κB positive feedback loop [31].

### Functional roles of mesothelin in tumorigenesis

The overexpression of MSLN across multiple cancer types has prompted studies aimed at evaluating its role in cancer development and progression. These studies have primarily highlighted MSLN’s involvement in the metastatic spread, progressively revealing the mechanisms underlying this process. Although the identification of a unified model describing the involvement of MSLN in cancer onset and progression remains challenging, additional evidence continues to emerge, gradually clarifying its biological significance.

#### MSLN regulates cell survival and proliferation

MSLN overexpression plays a central role in initiating multiple signaling events that collectively promote cell survival and proliferation under both anchorage-dependent and independent conditions. These studies have been conducted primarily in pancreatic cancer. One study demonstrated that aberrant MSLN expression enhances activation of the transcription factor Stat3, which in turn upregulates cyclin E and CDK2, thereby driving S-phase progression [32, 33]. Other researchers reported that MSLN also supports proliferation under conditions in which cells should undergo anoikis, specifically upon loss of anchorage [34]. This anchorage-independent survival is associated with ERK1/2 activation and suppression of the pro-apoptotic protein Bim.

A subsequent study by Bharadwaj and colleagues revealed that MSLN overexpression activates NF-κB signaling, which in turn promotes the production of high levels of IL-6 and soluble IL-6 receptor (sIL-6R) [35]. This enhances IL-6 trans-signaling and drives cell survival, proliferation, and spheroid formation. Shortly thereafter, another study added an additional layer to this pathway, showing that MSLN confers strong resistance to TNF-α-induced apoptosis and growth inhibition (Fig. 5) [30]. MSLN achieves this by inducing constitutive NF-κB and Akt activation, which increases IL-6 and Mcl-1 expression and supports cell survival and entry into the S-phase. These findings define an MSLN–NF-κB–IL-6–Stat3–Mcl-1 (and Akt) survival axis that protects cancer cells, particularly in inflammatory conditions. Another study investigating MSLN’s role in survival and proliferation showed that MSLN enhances anti-apoptotic proteins (Bcl-2, Mcl-1) and suppresses the pro-apoptotic Bax [36]. Importantly, MSLN drives proliferation through both p53-dependent and p53-independent mechanisms: when p53 is present, MSLN downregulates p53 and its downstream effector PUMA, whereas in p53-deficient cells, MSLN modulates the Bcl-2/Bax balance.

A recent work identified an MSLN-associated network module through histoepigenetic analysis [37]. In particular, the authors found that the expression of retinoic acid receptor gamma (RARG) and tyrosine kinase non receptor 2 (TNK2) was strictly dependent on the presence of MSLN. Both proteins are known to activate the aforementioned Akt pathway to support entry into the S-phase.

#### Mesothelin-driven metastatic spread

Several studies have reported an interaction between MSLN and CA125, an antigen expressed in various cancers as well as in normal peritoneal tissue. These findings provide strong evidence that MSLN-CA125 binding facilitates peritoneal dissemination in mesothelioma, ovarian, pancreatic, and gastric cancers [3]. More recently, Rupert and colleagues employed crystallographic analysis to elucidate the molecular details of this interaction, revealing that MSLN binds to specific Sea urchin sperm protein, Enterokinase, and Agrin (SEA) modules of CA125, independent of glycan involvement [38]. This study also highlights how the tandem arrangement of SEA modules enhances clustering of membrane MSLN and sMSLN, thereby influencing MSLN signaling. Supporting this, 3T3 cells transfected with a mesothelin expression vector exhibited increased adhesion to culture dishes compared to the wild-type cells [39], suggesting that MSLN may contribute to cell-to-cell and ECM adhesion. These findings imply a structural role for MSLN in facilitating intercellular interactions and anchoring within the TME.

Similar findings were reported in ovarian [40] and pancreatic cancers [41], underscoring the involvement of MSLN.

Ovarian cancer (OC) can disseminate into the peritoneal cavity by shedding cancer cells from the primary site. These cells are often found as multicellular heterotypic aggregates composed of OC and mesothelial cells. While the formation of such aggregates depends on the presence of mesothelial cells, it appears to be independent of MSLN expression. However, higher MSLN levels were associated with the formation of larger aggregates, which exhibited increased resistance to chemotherapeutic agents [40].

A similar pattern of behavior has been observed in PDAC, where knocking out MSLN prevents the formation of heterotypic aggregates and the peritoneal dissemination of cancer cells. Comparable outcomes were reported when MSLN was mutated to prevent its interaction with CA125, further underscoring the importance of this molecular interaction in facilitating peritoneal metastasis [41]. In this context, sMSLN seems to play a dual role in PDAC. On one hand, exposure of PDAC cells to sMSLN activates inflammation-related pathways, culminating in increased IL-1α secretion that may promote the metastatic phenotype. On the other hand, an excess of sMSLN inhibits cellular aggregation and metastatic spread by competing with membrane-bound MSLN for the interaction with CA125 [41]. Interestingly, the capability of MSLN to promote the peritoneal dissemination of primary cancers may involve mechanisms beyond its interaction with CA125. Hilliard and colleagues reported that MSLN affects the collagen structure of the peritoneal basal membrane, possibly influencing its permeability to cancer cells [40]. To evaluate the involvement of MSLN in this aspect, the authors compared the peritoneal ultrastructure of MSLN^KO^ mice with that of MSLN^WT^. The MSLN^KO^ mice presented omental tissues with more prominent long and thick collagen banding and with smaller fenestrations that, overall, covered less area. Additionally, the authors observed a reduced degree of fibril orientation that may be responsible for the thick collagen structure [40].

**Fig. 4:**
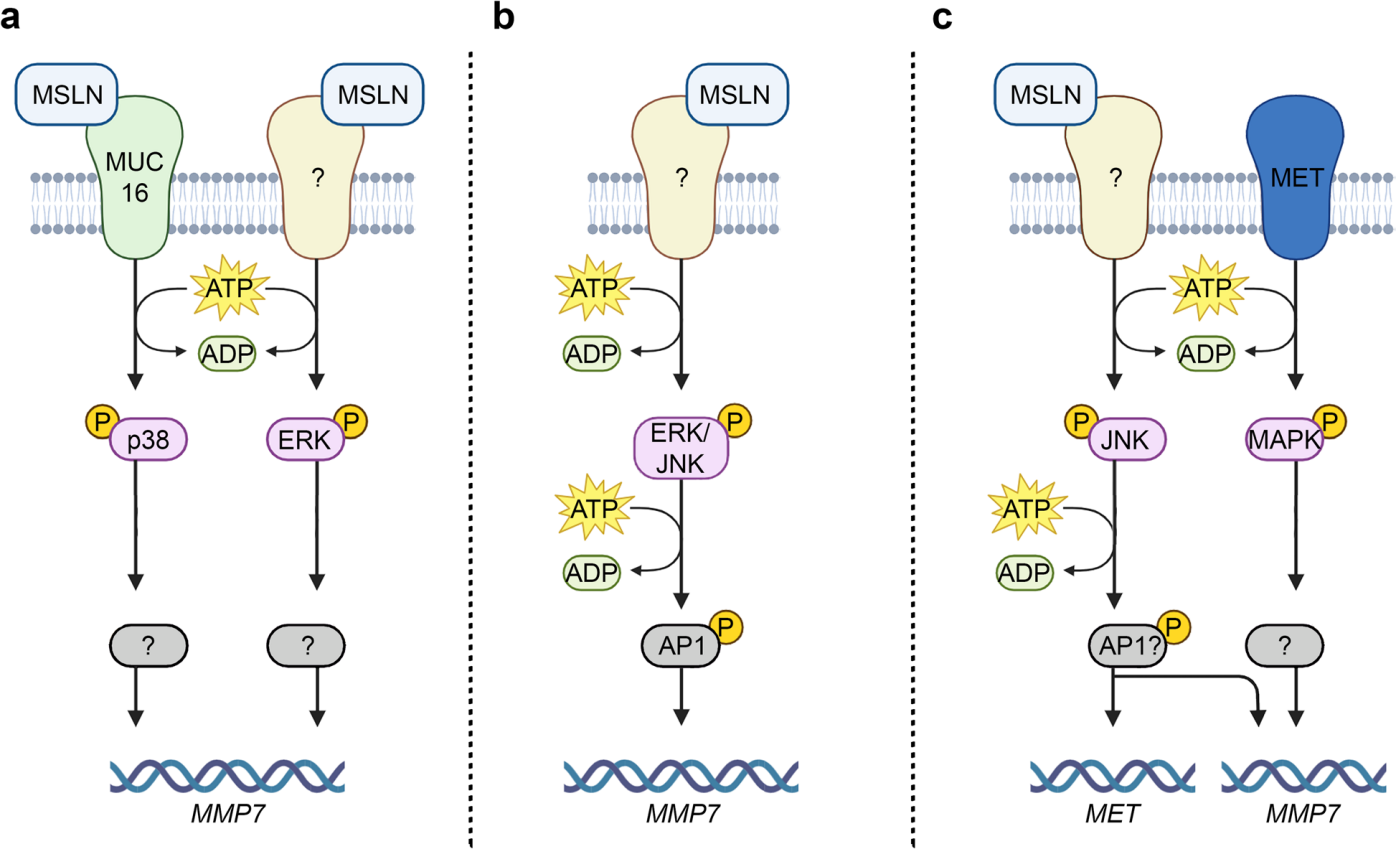
Pathways through which MSLN leads to an enhanced metastatic phenotype in pancreatic ductal adenocarcinoma (a), ovarian cancer (b), and non-small cell lung cancer (c). In each of these cancer types, MSLN stimulates the phosphorylation of MAP kinases, including p38, ERK, and JNK, which, in turn, leads to transcriptional upregulation of the *MMP7* gene. Evidence from ovarian cancer and non-small cell lung cancer suggests that AP1 could be the transcription factor involved in MSLN-related transcriptional activation of *MMP7*

Beyond mediating cancer cells anchorage to peritoneal tissues and promoting heterotypic aggregate formation, the MSLN-CA125 interaction appears to activate intracellular signaling pathways that drive metastatic dissemination. Chen and colleagues observed that exposing pancreatic cancer cells to MSLN stimulated the phosphorylation of the Mitogen-Activated Protein Kinase (MAPK) p38 in a CA125-dependent fashion. The activation led to upregulation of the *matrix metalloproteinase 7* (*MMP7*) gene, enhancing the invasive potential of the cells [42] (Fig. 4a). Interestingly, p38 activation was not the only way through which MSLN affected the expression of *MMP7*. Indeed, following MSLN exposure, the authors also observed a CA125-independent phosphorylation of other MAPK, ERK1/2, that increased the expression of *MMP7* [42] (Fig. 4a).

Similar mechanisms have been described for other cancers. In a study conducted on OC, the authors found that silencing *MSLN* in OVCAR3 cells reduced MMP-7 expression, and silencing *MMP7* negatively affected the migratory and invasive ability of these cells [43]. Conversely, treatment of MSLN-negative cells with recombinant sMSLN led to increased expression of MMP-7 and enhanced metastatic behavior. When examining the changes in the phosphorylation status of the MAPK proteins, the authors found that the MSLN exposure led to increased phosphorylation of ERK1/2 and JNK and that this phosphorylation significantly affected the MMP-7 levels. Interestingly, the authors also provided evidence that the transcription factor AP1 was involved in the transcriptional activation of the *MMP7* gene following MSLN stimulation and MAPK activation [43] (Fig. 4b). The involvement of AP1 in regulating the expression of *MMP7* was not surprising, as AP1 is a known downstream transcription factor in the MAPK signaling, and its binding to the *MMP7* promoter was previously described by Yamamoto and colleagues in 1995 [44].

Another piece of evidence supporting the relationship between MSLN and MMP-7 and their involvement in cancer metastasis comes from a study conducted on NSCLC [45]. In this recent study, Xia and colleagues reported that MSLN facilitated the establishment of brain metastasis in NSCLC by activating the MAPK JNK, leading to the degradation of the tight junctions that constitute the blood–brain barrier (BBB) [45]. The authors observed that the ability of the brain metastatic cells PC9-BrM to penetrate the BBB was significantly reduced upon MSLN silencing (shMSLN). Moreover, exposing an *in vitro* model of BBB to the PC9-BrM conditioned medium caused a reduction of JAM-A, VE-cadherin, and claudin-5, which ultimately led to the disruption of the barrier. Notably, this effect was not observed when the medium was derived from the shMSLN PC9-BrM cells, suggesting that MSLN plays a role in this process [45]. The authors provided evidence that MSLN enhances the activating phosphorylation of JNK, which correlates with increased expression and secretion of MMP-7. Although this study does not provide direct evidence for AP1 involvement, it is plausible to hypothesize that AP1 may contribute to *MMP7* transcription following MSLN stimulation, a mechanism previously reported in OC [43] (Fig. 4c). Additionally, the activation of JNK pathway has been shown to upregulate the expression of MET (also known as hepatocyte growth factor receptor, HGFR), which plays a critical role in the acquisition of a metastatic phenotype. Inhibition of MET in PC9-BrM cells reduced their ability to penetrate the BBB, while MET overexpression restored the invasive phenotype in shMSLN cells (Fig. 4c). Notably, similar to MSLN, MET expression affected the capability of conditioned media to disrupt the BBB integrity [45].

The identification of an AP1 binding site within the core promoter of *MET* [46] supports the hypothesis that MSLN may induce *MET* transcription via the activation of JNK and its downstream effector, the transcription factor AP1. MET, in turn, has been shown to stimulate the transcription of *MMPs* through the MAPK signaling cascade [47]. These findings suggest that, in NSCLC, MSLN promotes the expression of both MET and MMP-7 through the JNK/AP1 axis. Moreover, MET further increases the expression of MMP-7, thereby reinforcing the oncogenic signaling network initiated by MSLN (Fig. 4c).

Although the mediators involved in MSLN signal transduction may vary in a cancer-specific manner, they appear to converge on a common downstream target: the *MMP7* gene. *MMP7* encodes a proteolytic enzyme known to facilitate cancer cell mobility and invasion [48]. Consistently with its functional role and further reinforcing its association with MSLN, elevated MMP-7 expression has been reported across several MSLN-positive malignancies, including pancreatic, breast, colorectal, ovarian, and NSCL cancers [42, 43, 49–51]. In addition to its association with tumor growth, migration, metastasis, and poor prognosis [48], MMP-7 exerts its oncogenic effects largely through degradation of the ECM, particularly the basement membranes [52]. These membranes serve as protective barriers composed predominantly of collagen, laminin, and proteoglycans, which prevent cancer cells from invading surrounding tissues. ECM degradation also results in the release of sequestered growth factors, such as Tumor Necrosis Factor alpha (TNF-α) and transforming growth factor beta (TGF-β), which further stimulate the proliferation of cancer cells [52].

Another route that MSLN activates to promote the metastatic spread is the stimulation of the EMT, a process through which epithelial cells lose their polarization and their interaction with the basement membrane and acquire a mesenchymal phenotype characterized by enhanced invasiveness and migratory abilities [53]. As such, EMT is considered an early event in cancer metastasis. In a study on mesothelioma and lung cancer cell lines, He and collaborators employed a PCR array approach to evaluate the changes in the expression of EMT-related genes caused by *MSLN* silencing [4]. The authors found that eight epithelial differentiation-related genes were upregulated (including *E-cadherin*), and six growth factor related genes were downregulated (including the EMT-related genes *SNAIL* and *TWIST*) following* MSLN* silencing, linking MSLN to the stimulation of the EMT process. Interestingly, occludin, a tight junction forming protein, was upregulated in *MSLN*-silenced cells (shMSLN), further supporting MSLN’s role in disrupting the tight junctions during the metastatic process. These gene expression changes were also accompanied by phenotypic alterations, with shMSLN cells forming smaller tumors and fewer metastatic nodules in vivo, compared with MSLN-expressing cells [4].

Similar results were reported for pancreatic cancer by Hu and colleagues [54]. In their work, the authors knocked out MSLN expression in ASPC-1 cells and observed an increase in the epithelial marker E-cadherin and a decrease in the expression of the mesenchymal markers Vimentin and Snail. Consistently, overexpressing MSLN in the MSLN-negative Mia PaCa-2 cells led to reduced E-cadherin expression and enhanced Vimentin and Snail expression [54].

**Fig. 5:**
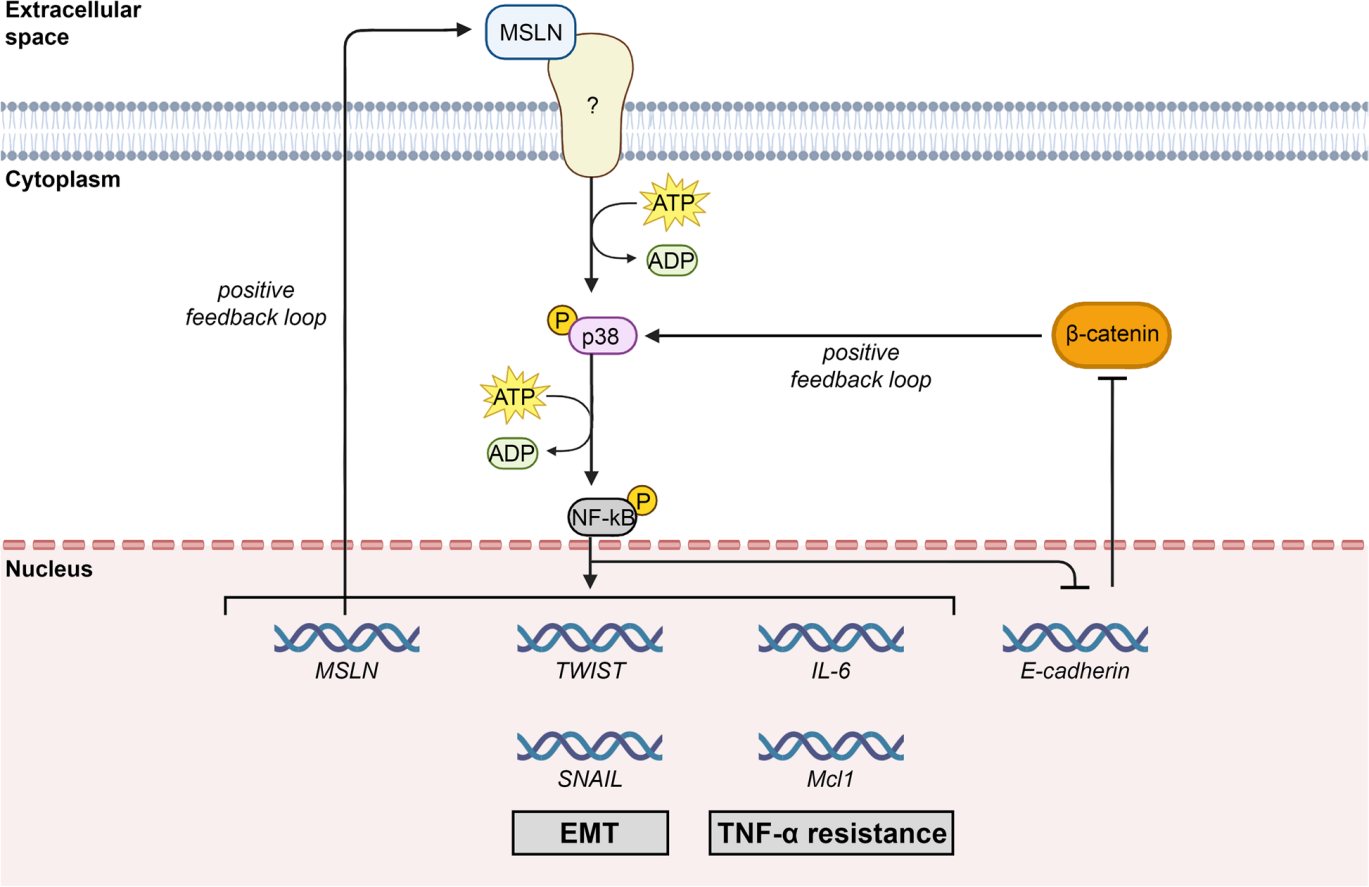
Schematic representation of the proposed mechanism linking MSLN to NF-κB. MSLN activates the NF-κB signaling pathway, which in turn activates MSLN gene expression in a positive feedback loop. Furthermore, NF-κB activation leads to the transcriptional upregulation of EMT-inducing genes SNAIL and TWIST. In malignant melanoma cells, activated NF-κB downregulates E-cadherin expression, resulting in an accumulation of free cytoplasmic β-catenin. This β-catenin pool subsequently triggers p38 MAPK activation, which further enhances NF-κB signaling, creating a positive feedback loop that sustains EMT progression. In pancreatic cancer, NF-κB induces the expression of IL-6 and Mcl1, which are genes associated with TNF-α apoptosis resistance

While these studies highlighted a role for MSLN in stimulating the EMT, no direct evidence exists concerning the exact pathways activated by MSLN to achieve this result. However, evidence from studies focused on the NF-κB signaling in the EMT process may allow some speculations. Indeed, in these studies, NF-κB was shown to be responsible for the up-regulation of *SNAIL* and *TWIST* genes. In malignant melanoma cells, NF-κB caused a reduction in E-cadherin expression, resulting in an increased amount of free cytoplasmic β-catenin, which in turn led to the p38 MAPK-mediated activation of NF-κB [55]. Although further studies are needed to evaluate this aspect, previous observations linking MSLN to the activation of NF-κB signaling in different contexts suggest that MSLN-mediated EMT could, at least in part, be driven by NF-κB-dependent up-regulation of *SNAIL* and *TWIST* [30] (Fig. 5). This highlights another role for NF-κB, which, as mentioned above, is also involved in resistance to TNF-α–mediated apoptosis, further underscoring its multifaceted role in tumor cell survival and adaptation.

#### Mesothelin-mediated remodeling of the tumor microenvironment

Increasing evidence shows the involvement of the TME in the onset, progression, and drug resistance of virtually every type of malignancy. While the composition and specific characteristics of TME vary across different types of cancer, general features may be recognized. TME is mostly characterized by the presence of cancer-associated fibroblasts (CAFs) and tumor-associated macrophages (TAMs), which engage in an intense crosstalk with cancer cells and other TME components, often promoting cancer growth and survival by secreting immunosuppressive molecules and activating pro-survival pathways.

Several studies have reported MSLN to be associated with specific features of the TME. For instance, Malla and colleagues observed that high MSLN expression in colorectal cancer (CRC) was associated with low levels of IFN-γ and high levels of HAVCR2 and PD-L1, accompanied by an increased infiltration of regulatory T cells (Tregs) in the TME [37]. Similarly, in PDAC, MSLN expression was associated with reduced infiltration of CD8^+^ T-cells, lymphocyte infiltration score, and cytolytic activity [56]. Additionally, soluble MSLN was reported to accumulate within the PDAC microenvironment, where it could act as a decoy for MSLN-targeting therapies, reducing their efficacy. This hypothesis stemmed from the observation that the PDAC patients with high MSLN expression in tumor biopsies showed unusually low levels of MSLN in the bloodstream, suggesting that shed MSLN was somehow retained within the TME. Although convincing experimental evidence was provided, the mechanisms behind this retention remain unclear and may be attributed to the ECM composition, rebinding of shed MSLN to CA125, or other unknown factors [42]. Although these studies suggested an association between MSLN and immunosuppressive features of the TME, opposite evidence was reported for other cancer types. For instance, in mesothelioma, a high MSLN expression was associated with an immunostimulatory TME characterized by increased infiltration of CD8^+^ T cells and CD68^+^ macrophages [57]. Additionally, high MSLN-expressing mesotheliomas showed increased presence of collagen type I fibers, forming a dense collagen matrix that acts as a physical barrier limiting cancer dissemination [38]. Interestingly, collagen type I expression has been functionally linked to MSLN also in a non-malignant context, where it contributed to the development of cholestatic liver fibrosis [39]. Specifically, it has been observed that the MSLN/CA125 complex may interact with Thy-1, promoting its dissociation from TGFβRI and the activation of this latter in the presence of a TGF-β stimulation. The activation of the TGF-β-Smad signaling resulted in the transcriptional activation of fibrogenic genes, including collagen type I, ultimately leading to the establishment of liver fibrosis [39, 40]. Although not directly transferable to malignancy, these observations, along with the well-established link between fibrosis and cancer, strongly support further research into the role of MSLN in this context [41]. An association between MSLN and specific features of TME has recently emerged in triple-negative breast cancer (TNBC) as well. In this case, the authors observed that MSLN-high TNBCs were characterized by increased stromal and leukocyte fraction and increased lymphocyte infiltration. Particularly, the authors observed a significantly increased infiltration of M1 macrophages, suggestive of a pro-inflammatory response. Of note, no association was observed with specific CD4^+^ or CD8^+^ lymphocyte infiltration [43].

**Fig. 6:**
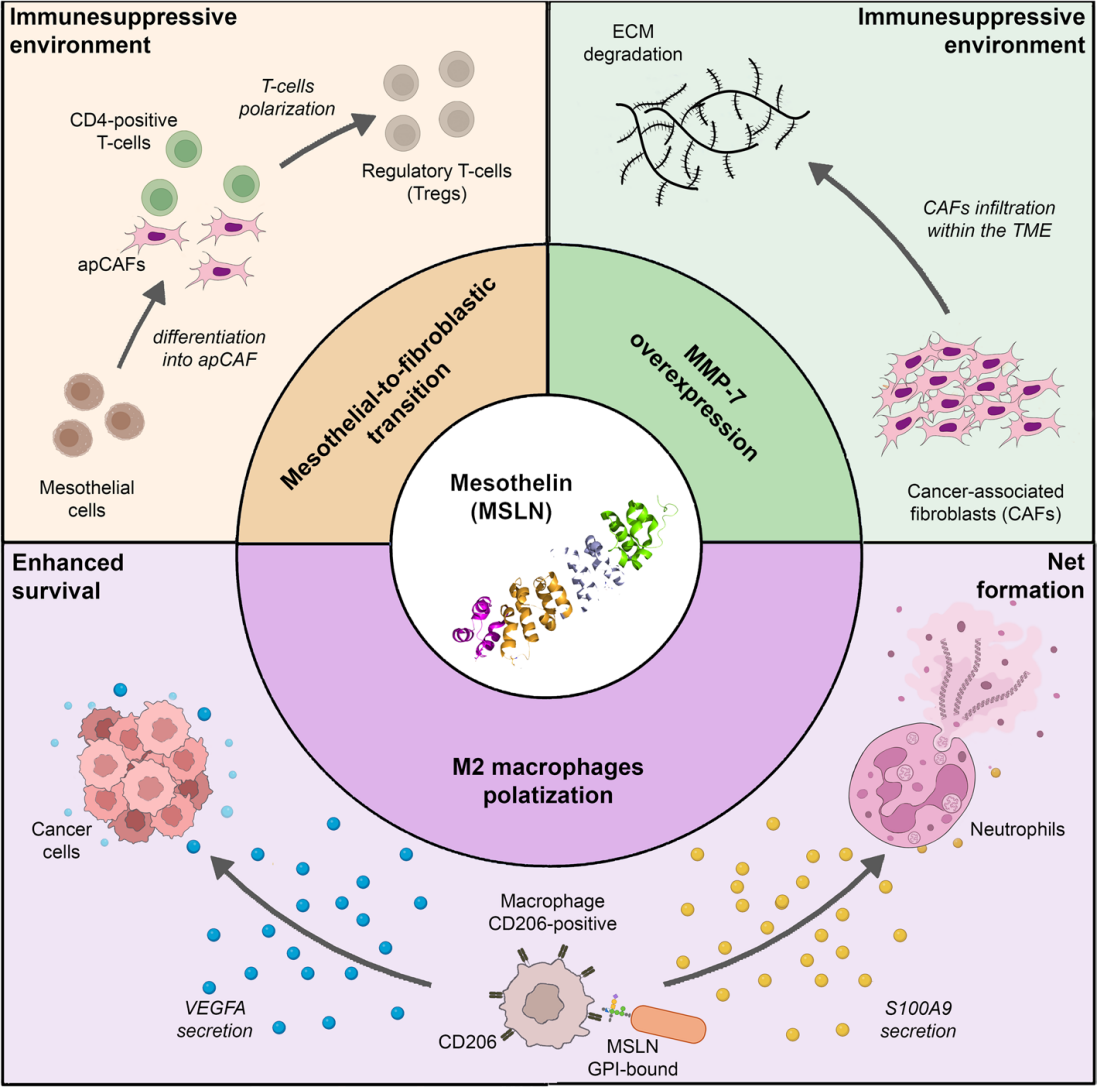
Schematic representation of the mesothelin-mediated tumor microenvironment (TME) remodeling through three main mechanisms that include the induction of the mesothelial-to-fibroblastic transition, the overexpression of the matrix metalloproteinase 7 (MMP-7), and the macrophage polarization towards an M2 phenotype. ApCAFs: antigen-presenting cancer-associated fibroblasts (apCAFs); TME: tumor microenvironment; NET: neutrophil extracellular traps

Beyond these correlative observations, emerging studies are beginning to elucidate how MSLN may mechanistically shape key features of the TME. MSLN has been reported to upregulate MMP-7 expression through the mechanisms described in the previous chapter. Since MMP-7 plays a central role in ECM remodeling and immune-cell recruitment [52, 58], this connection provides a plausible link between MSLN and TME modulation. Accordingly, a pan-cancer transcriptomic analysis recently revealed a positive association between *MMP7* expression and the infiltration of CAFsand myeloid cells across most tumor types, suggesting a broad involvement of MMP-7 in shaping the stromal and immune landscape [59]. Consistently, mechanistic evidence in colorectal cancer indicates that MMP-7 can promote macrophage polarization toward an M2 phenotype via the miR-411-3p axis [60]. Specifically, miR-411-3p was found to negatively regulate MMP7, thereby limiting M2 macrophage polarization. Overexpression of this miRNA reduced the expression of the M2 markers CD206 and ARG1, whereas its inhibition restored MMP-7 levels and enhanced the same markers [60]. These findings support a mechanistic model in which MSLN-driven *MMP7* upregulation could contribute to an immunosuppressive TME by promoting macrophage polarization toward an M2-like phenotype. In a PDAC study, the authors identified a subset of CAFs originating from mesothelial cells and characterized by MHC class II expression (antigen-presenting CAFs, apCAFs) [61]. Within the TME, interactions between apCAFs and CD4⁺ T-cells promoted the acquisition of Treg markers, CD25 and FoxP3. The authors proposed that apCAFs facilitate T-cell polarization toward an immunosuppressive Treg phenotype due to their lack of co-stimulatory molecules. Importantly, inhibition of MSLN with a monoclonal antibody reduced the mesothelial-to-apCAF transition and consequently decreased Treg polarization [61], indicating an active role for MSLN in shaping an immunosuppressive axis of the TME. Interestingly, the mesothelial-to-fibroblastic transition giving rise to apCAFs closely resembles mesothelial activation and ECM deposition seen in fibrotic processes [62, 63], suggesting that similar mesothelin-dependent mechanisms might be at play. The most direct evidence of MSLN involvement in shaping the TME comes from a recent study by Luckett et al. [64]. Using both in vitro and in vivo approaches, the authors showed that MSLN secreted by PDAC cells promotes the recruitment and polarization of CD206⁺ macrophages. Previous work indicated that, through the mannose residues on its GPI anchor, MSLN can bind to CD206 receptors on the surface of macrophages [20]. Based on this, the authors hypothesized that a similar interaction mediates the recruitment of CD206^+^ macrophages observed in vivo. Supporting this mechanism, PDAC cells were found to overexpress GPLD1, a phospholipase responsible for releasing GPI-anchored MSLN [20]. Importantly, these CD206⁺ macrophages produced soluble factors including arginase 1 (ARG1), vascular endothelial growth factor A (VEGFA), and the S100 calcium-binding protein A9 (S100A9), which are known mediators of TME remodeling. In this context, the macrophage-secreted VEGFA was shown to bind the VEGF receptor on PDAC cells, promoting their survival and growth, while no effect was observed on the angiogenesis within the TME. S100A9 was also identified as a direct mediator of MSLN-driven TME modulation. At metastatic sites, particularly in the lungs, high S100A9 levels correlated with increased neutrophil infiltration and enhanced formation of neutrophil extracellular traps (NETs) [64]. NETs are web-like structures composed of decondensed chromatin and granule proteins released by neutrophils that facilitate metastasis through both structural and cell-intrinsic mechanisms. Structurally, NETs form scaffolds around circulating tumor cells, promoting their survival at metastatic sites. Additionally, NETs can enhance cancer cell metabolism by increasing mitochondrial ATP production and can activate signaling pathways that promote tumor cell motility [65]. Another mechanism through which MSLN promotes immune evasion relies on the Wnt/b-catenin axis. Recently, Zhong et al. reported that organoids derived from 28 high-grade serous ovarian cancer (HGSOC) patients displayed different grades of sensitivity to anti-PD-1 treatment ex vivo that correlated with MSLN expression [66]. Specifically, responsive organoids (N = 6) showed low MSLN expression, and an abundance of cytotoxic CD8 + T-cells and tumor-suppressive TAM expressing high HLA-DR, CD86, and CXCL9. On the other hand, resistant organoids (N = 22) expressed high MSLN and were characterized by an immune-suppressive TME with high Tregs abundance and a CD206 + TAM polarization. Interestingly, in vitro silencing of MSLN resulted in a transition from the immune-suppressive TME (Tregs and CD206 + TAM) to the tumor suppressive one (CD8 + T-cells and CD86 + TAM), suggesting the MSLN functional involvement in this process. Mechanistically, MSLN promoted the expression of CD24 by activating the Wnt/β-catenin pathway, and CD24 drove the immune-suppressive polarization of the TME likely through the CD24/Siglec-10 axis. Consistently, forced CD24 overexpression was able to rescue the aggressive cancer phenotype in the MSLN-silenced cells, reestablishing the immune-suppressive TME and promoting immune evasion [66]. Overall, the emerging data suggest a direct involvement of MSLN in shaping the TME toward pro-tumorigenic features, including the induction of immunosuppressive phenotypes in macrophages and T-cells, activation of pro-survival and motility pathways, and promotion of NETosis within the TME. The mechanisms through which MSLN can reshape the TME are summarized in (Fig. 6).

## Mesothelin as a diagnostic and prognostic biomarker

Given its overexpression in many cancer types, MSLN has been widely investigated as a potential diagnostic and prognostic biomarker for these malignancies. Among the MSLN-overexpressing cancers, PM is the one with the greatest need for new diagnostic tools. Indeed, the diagnosis of PM is quite complex due to the long latency between asbestos exposure -the main etiological factor—and the onset of the disease, the mild symptoms characterizing the initial stages, and the lack of minimally invasive diagnostic tools suitable for health surveillance programs [67]. To date, the diagnosis of PM largely relies on the histopathological assessment of invasive pleural biopsies and often occurs at late stages [68]. Circulating MSLN has been widely evaluated as a diagnostic biomarker for PM and tested across different matrices (*serum*, plasma, and pleural fluid) and patient/control groups. Overall, the accuracy of MSLN does not appear to be affected by the matrix used for analyses. In a recent meta-analysis evaluating the diagnostic performance of MSLN across 56 studies, Schillebeeckx et al. reported a pooled area under the curve (AUC) ranging from the minimum value of 0.81 (0.79–0.83) when MSLN was measured in the *serum*, to a maximum of 0.86 (0.79–0.92) in the plasma, with *serum* being the most commonly used matrix [69].

Studies conducted on *serum* MSLN reported optimal cut-off values ranging from 0.55 nM to 2.4 nM [69]. The highest diagnostic accuracy was reported by Napolitano et al. when evaluating 22 PM patients and 20 individuals with a history of asbestos exposure [70]. In this context, the authors reported an AUC of 0.93 (0.86–1.00). Similar results were also reported by Sriram et al. who compared 16 PM patients with 23 subjects with benign pleural effusions (BPE) [71]. Specifically, the authors reported an AUC of 0.94 (0.87–1.00) with a sensitivity of 75% and a specificity of 90% at a cut-off of 1.34 nM. Although these results suggest high diagnostic accuracy, other studies have reported different findings. In the context of an early diagnosis, the most interesting comparison is probably between early-stage mesothelioma and asbestos-exposed individuals. To the best of our knowledge, only one study has carried out this kind of comparison. In their work, Pass et al. reported an AUC of 0.81 (0.75–0.87) and a sensitivity and specificity of 60% and 89% respectively, when a cut-off of 1.9 nM was used [72]. However, the authors also observed a significant difference in the *serum* concentration of MSLN between stage 1 (2.09 ± 0.41 nM) and stage 2–4 (10.61 ± 3.89 nM) mesotheliomas. Consistently, when the ROC curve analysis was restricted to stage 1 PM and asbestos-exposed individuals, the AUC dropped to 0.74 (0.64–0.84) with specificity increasing to 91% but the sensitivity dropping to 58% at a cut-off value of 2.0 nM, highlighting the questionable utility of MSLN in the context of an early diagnosis [72].

Most studies agree, to some extent, on the diagnostic potential of MSLN, but variability in the cut-off values used and the reported accuracy remains a major obstacle to fully understanding its diagnostic relevance. This inter-study variability may be partly due to the limited number of PM patients enrolled in most of the studies and, to a larger extent, on the heterogeneity of the control groups, which range from healthy individuals without a previous asbestos exposure, to asbestos-exposed patients affected by various benign respiratory diseases (BRD) [69]. Although further studies may help clarify its true diagnostic utility, the available data indicate that MSLN has good specificity but lacks the sensitivity required for a stand-alone biomarker [69].

Over the years, other biomarkers have been combined with MSLN to develop a diagnostic panel aimed at enabling early detection of PM in at-risk populations while minimizing false-positive results. With this aim, most studies have evaluated the sensitivity of these panels at fixed specificity thresholds. In this respect, Johnen et al. evaluated the diagnostic properties of MSLN, calretinin, and their combination in a prospective study that included 31 PM patients and 136 asbestos-exposed controls [73]. Interestingly, the 31 PM samples were pre-diagnostic, collected from 1 to 44 months prior to the diagnosis. When evaluated as stand-alone biomarkers, MSLN and calretinin yielded AUCs of 0.66 (0.55–0.78) and 0.74 (0.63–0.85), respectively, with sensitivities of 19% at a fixed specificity of 99% and 26% at a fixed specificity of 98% [73]. However, when MSLN and calretinin were combined, the AUC increased to 0.83 (0.65–0.83) and the sensitivity at a fixed specificity of 98% rose to 39%. Notably, restricting the analysis to samples collected between 1 and 15 months (N = 26) or between 1 and 6 months (N = 9) before the diagnosis resulted in the sensitivity increasing to 46% and 56%, respectively [73]. Although the sensitivities obtained were still too low for clinical application, Johnen’s group was, to the best of our knowledge, the only one to use pre-diagnostic samples to evaluate the accuracy of combined biomarkers. Other groups have evaluated the combination of MSLN with additional markers, but only in case–control studies with limited clinical impact.

Another way to improve the performance of a diagnostic biomarker is to account for confounding factors that could alter its blood concentration. In this respect, we recently analyzed the functional role of four single-nucleotide polymorphisms (SNPs) within the proximal promoter of *MSLN* (rs3764247 A > C, rs3764246 A > G, rs2235503 C > A, rs2235504 A > G) [74]. Using a fluorescent reporter-based assay, we showed that the variant allele (A) of rs2235503 enhanced *MSLN* expression and, consistently, was associated with increased *serum* MSLN concentration. When we evaluated its diagnostic performance in a cohort of 72 PM and 613 controls, MSLN yielded an AUC of 0.87 with a sensitivity of 77.78% and a specificity of 79.91% at a 1.28 nM cut-off. However, when the population was stratified based on the rs2235503 genotype (CC or AC + AA), the AUC for the CC group increased to 0.92, with a sensitivity of 84.31% and a specificity of 82.86% at a 1.12 nM cut-off. Conversely, the AUC for the AC + AA group dropped to 0.80, with a sensitivity of 52.58 but a specificity of 96.69 at a 3.092 nM cut-off value [74]. These observations suggested that genotype-specific thresholds should be evaluated when using MSLN as a diagnostic biomarker in PM to maximize its accuracy.

In a similar study, Goricar et al. identified another SNP, rs1057147 G > A, affecting MSLN *serum* levels [75]. The A variant was associated with increased MSLN concentration and, when genotype was considered, specificity increased from 0.88 in the general population to 0.91 (for the GG genotype), 0.90 (for the GA), and 0.93 (for the AA). In line with our observations, the AUC increased from 0.80 in the general population to 0.83 in the GG group but decreased to 0.76 and 0.78 in the GA and AA groups, confirming that those alleles associated with enhanced MSLN expression reduce the diagnostic performance [75].

Recently, Rihs et al. examined the role of these SNPs in a German cohort comprising 102 PM and 410 BRD patients [76]. Consistent with previous results, they reported that both the rs2235503 and rs1057147 variant alleles were associated with increased *serum* MSLN. Regarding rs2235503, at the optimal cut-off established for the general population, the authors observed that the risk of false positives was 3.7-fold and 20.7-fold higher in individuals carrying the CA and AA genotypes, compared with the CC homozygous. Similarly, carrying the A variant of rs1057147 showed a 4.08-fold increase in false positives, further supporting the need for genotype-adjusted cut-off values [76].

In contrast to the extensive body of research on MSLN as a diagnostic biomarker for PM, only a limited number of studies have investigated its role in other MSLN-expressing cancers. Most of them can be effectively summarized through existing meta-analyses. A meta-analysis by Zhu et al. pooled data from 12 studies involving 928 PDAC patients [77]. These studies evaluated MSLN positivity in samples obtained via surgical resection or fine-needle aspiration. However, the cut-off methods and thresholds varied across studies, contributing to substantial heterogeneity in the results. Reported sensitivity values ranged from 0.68 (0.43–0.87) [78] to 0.97 (0.87–1.00) [79], while specificity ranged from 0.33 (0.10–0.65) [80] to 1.00 (0.91–1.00) [81]. The pooled sensitivity of 0.71 (0.67–0.75) and specificity of 0.88 (0.85–0.91) suggest that MSLN holds promise as a biomarker for the histological diagnosis of PDAC. Nonetheless, as with PM, strategies to enhance its sensitivity may be necessary. Further studies are warranted to strengthen the evidence supporting its diagnostic utility.

Similar findings have been reported in OC based on 12 studies analyzed by Madeira et al. [82] encompassing a total of 1561 individuals. As for PM and PDAC, OC also exhibited considerable heterogeneity regarding optimal cut-off values, as well as reported sensitivity and specificity. Specifically, sensitivity ranges from 38% (29%−47%) [83] to 97% (86%−100%) [84], while specificity varied between 54% (33%−74%) [85] and 100% (92%−100%) [86]. When pooled values were calculated, the sensitivity and specificity were reported as 62% (58%−66%) and 94% (92%−95%), respectively [82].

Data from PDAC and OC cohorts reinforce the observation that low sensitivity remains the most significant limitation of MSLN as a diagnostic biomarker. While studies involving pre-clinical samples would be valuable for better characterizing MSLN in clinically relevant settings, efforts should also focus on enhancing its sensitivity, especially for early cancer detection. Potential strategies may include accounting for confounding factors and exploring the combined use of MSLN with other biomarkers.

Since MSLN has been shown to play an active role in cancer progression, particularly in metastatic dissemination and in promoting the establishment of an immunosuppressive TME, its prognostic value has also been investigated. In this context, a meta-analysis summarizing findings in PM is available [87]. This meta-analysis included 7 studies that assessed *serum* MSLN levels and one study that measured MSLN concentration in pleural fluid. A certain degree of heterogeneity was observed for the cut-off values, ranging from 1 to 5 nM in *serum* and being 26 nM in pleural fluid. Five of the seven *serum*-based studies reported a significant association between elevated MSLN levels and poor prognosis, with Hazard Ratios (HR) ranging from 1.62 (1.10–2.39) [88] to 5.99 (1.76–20.40) [89]. The remaining two studies showed a similar trend but did not reach statistical significance [90, 91]. Overall, the pooled HR was 1.958 (1.531–2.504), with a p-value < 10^–3^ [87].

For other major MSLN-expressing malignancies, evidence regarding the prognostic role of MSLN remains limited, and to the best of our knowledge, no meta-analysis on this topic is currently available. Moreover, existing results are often inconsistent, contributing to a lack of consensus on the prognostic significance of MSLN. A recent review by Giordano et al. highlighted that, in OC, the prognostic utility of MSLN is highly dependent on the biological matrix used for its assessment [92]. Indeed, Okła et al. reported that MSLN levels measured in both plasma and tumor tissues were positively correlated with cancer stage and grade. Conversely, this correlation was not observed for MSLN levels measured in peritoneal fluid. Moreover, the authors reported that elevated plasma MSLN levels in preoperative patients were indicative of a poorer prognosis, which was associated with a reduced 5-years overall survival (OS) [93]. Similar findings were also reported by Wu et al. in a cohort of 78 epithelial ovarian cancer (EOC) patients [94]. In this study, the authors observed a positive correlation between *serum* MSLN levels and cancer stage, as well as postoperative decrease in MSLN concentrations. These results suggest that MSLN may serve as a useful biomarker for monitoring disease progression and evaluating treatment response [94].

Many studies have evaluated the tissue MSLN expression as a potential prognostic factor; however, the findings remain conflicting, making it difficult to establish a clear consensus. In particular, Cheng et al. [95] and Yildiz et al. [96] reported that high MSLN expression in tumor tissues was significantly associated with poorer progression-free survival (PFS) and OS in patients with EOC. In contrast, Magalhaes et al. [97] found no significant association, while Yen et al. [98] observed an association between elevated MSLN levels and favorable prognosis [92]. In contrast, findings in PDAC suggest a different pattern: serum MSLN does not appear to provide prognostic indications, whereas tissue MSLN expression shows greater potential. Elevated tissue MSLN levels have generally been associated with poorer PFS and OS. However, the current evidence remains limited, preventing definitive conclusions on its prognostic value [99].

## Mesothelin targeted therapies

Given its overexpression in numerous solid tumors and its involvement in cancer progression, MSLN represents an attractive therapeutic target. The first attempt to explore the potential of MSLN-targeted therapy was carried out by Hassan and colleagues in 1999, using mice bearing MSLN-positive tumors [100]. In this study, the anti-MSLN K1 antibody was conjugated with indium^111^ (In^111^) and administered intravenously to assess its biodistribution. In^111^-K1 predominantly accumulated in MSLN-positive tumor masses, highlighting the potential of MSLN as a tumor-specific marker.

In addition, MSLN was also studied as a therapeutic target by conjugating the K1 antibody with a *Pseudomonas* exotoxin [101], leading to encouraging results for MSLN-targeted therapeutics. According to recent findings, targeting MSLN could enhance the efficacy of cytotoxic drugs and immune cell engagement, as emerging studies have identified MSLN as a modulator of the TME [61, 102, 103]. Thus, MSLN could function both as a passive therapeutic target and as an active effector influencing molecular interactions within the TME.

Building on this evidence, multiple strategies, ranging from protein-based to cell-based approaches, have been developed. These will be discussed in the following sections, highlighting how the cancer treatment landscape is evolving to address MSLN-specific delivery challenges in the dense stroma of solid tumors and the immunosuppressive conditions of the TME.

### Recombinant immunotoxins

After establishing the role of MSLN as a potential cancer antigen with the K1 antibody [48], the first MSLN-targeted cancer therapies employed proteins or Abs conjugated with cytotoxic molecules. These strategies offer the advantage of delivering anti-tumor drugs directly into cancer cells, thereby ensuring a tumor-specific targeting and minimizing the off-target effects commonly associated with systemic therapies [104]. To achieve effective tumor-specific drug action, protein-based therapies require internalization of the drug carrier into cancer cells following MSLN binding [105]. Immunotoxins fall within this therapeutic category. They consist of Ab fragments or protein-based antigen ligands fused to toxin proteins, typically derived from bacteria or plant sources. Among anti-MSLN immunotoxins, SS1P was the first therapeutic agent to enter clinical evaluation, advancing to Phase II clinical trials [9] (NCT01445392, NCT01362790, NCT00006981) (Table 1). SS1P combines a MSLN-targeting Ab variable fragment (Fv) with a portion of *Pseudomonas* exotoxin A (PE38). The anti-MSLN Fv ensures tumor-specific targeting, while PE38 exerts its cytotoxic effect (blocking protein synthesis and inducing apoptosis) upon internalization into MSLN-overexpressing cancer cells. SS1P demonstrated potent anti-tumor efficacy in both preclinical and clinical settings, including when administered in combination with standard chemotherapeutics [8, 9, 122–124]. However, SS1P and other PE38-based immunotoxins have also been observed to promote off-tumor toxicity, immunogenicity, and quick development of anti-drugs Abs (ADA) [125]. In recent years, immunotoxins design has evolved to enable tumor-specific activation of the toxin, aiming to reduce the side effects. One strategy involves engineering the toxin portion to remove epitopes recognized by T or B cells. In this context, Hollevoet and colleagues developed PE24, a toxin derived from PE38 with mutated B-cell epitopes [126]. Then, LMB-100 (also known as RG7787) was generated by fusing an anti-MSLN Fab to PE24, retaining the same anti-tumor effectiveness as SS1P but with reduced side effects [126, 127]. This novel recombinant immunotoxin progressed to phase I and II clinical trials, where it showed limited efficacy as a monotherapy. Most patients developed ADAs, reducing LMB-100 levels in blood, and dose-limiting toxicity was observed in 20% of patients. (NCT02317419, NCT02798536) (Table 1) [106]. To reduce ADAs formation, LMB-100 was also tested in combination with the enzyme inhibitor (tofacitinib) (NCT04034238). In phase I/II studies, patients with high MSLN-expressing tumors treated with a combination of LM-B100 and programmed death-ligand 1 (PD-L1) blocking agents (NCT02798536) [128] demonstrated poor clinical efficacy and unfavorable safety profile, with most AEs attributed to the anti-PD-L1 agent. LMB-100 combined with nab-paclitaxel (NCT02810418) (Table 1) [108] showed better tolerability accompanied by the stimulation of CD4 and CD8 antitumor cells. Clinical activity (partial response) was observed in patients who experienced side effects such as capillary leak syndrome. In accordance with these results, further improvements are necessary for PE24-based drugs. Recently, the PE24 toxin has been conjugated with an anti-MSLN nanobody (Nb, known as A1). The Nb scaffold (~ 15 kDa) is a single domain antibody derived from the variable fragment of heavy chain-only Abs in camelids, and it is significantly smaller than conventional therapeutic mAbs (~ 150 kDa). This reduced size may allow for faster systemic clearance and improved penetration into solid tumors [105]. Wang and colleagues further enhanced this construct by coupling the A1-PE24 immunotoxin with an additional Nb targeting human serum albumin (HSA), resulting in the Nb_HSA_-A1-PE24 complex [129]. Binding to HSA is essential for extending the immunotoxin’s half-life, thereby potentially increasing its therapeutic efficacy. To further refine tumor specificity, a cleavable linker (uPA) was used to link the Nb_HSA_ to A1-PE24 conjugate. In this way, the off-target toxicity and immunogenicity were reduced because the cleavage and activation of A1-PE24 immunotoxin from the Nb_HSA_ occurs only in the TME. Conditional activation of A1-PE24 demonstrated promising anti-tumor effects in pancreatic and gastric cancer xenograft models [129].

**Table 1:** MSLN-targeting strategies evaluated or under evaluation in clinical trials

Strategy	Study ID^a^	Intervention	Condition	Phase	Primary outcome measures^b^	Status—Main results^b^	References
Immunotoxin	NCT00006981	Continuous infusion SS1P	MSLN-positive cancers	1	Safety, MTD	Completed (2009)—SD (1 pt)	[9]
	NCT01445392	Multicycle SS1P + Pemetrexed + Cisplatin	Mesothelioma	1	Safety, MTD, Best OR	Terminated (2016)	
	NCT01362790	SS1P + Pentostatin + Cyclophosphamide	Mesothelioma	1/2	Response Assessment, SS1P antibody formation, AEs, RP2D	Completed (2016)—PR (2 pts); SD (24 pts); PD (10 pts)—AF (18 pts)—OS (4.2 −29.3 months)	
	NCT02317419	LMB-100 + gemcitabine + nab-paclitaxel	MSLN-positive cancers	1	Safety, ADAs, abnormal findings on physical examination, infusion-related reactions	Terminated because risk ratio did not justify continuing dosing patients (2015)	[106]
	NCT02798536	LMB-100/nab-paclitaxel	Mesothelioma	1/2	RP2D, ORR	Completed (2022)—no major response detected, ADAs 90% pts	[106]
	NCT04034238	LMB-100 + tofacitinib	MSLN-positive cancers	1	MTD, plasma drug levels, Aes	Completed (2021)—75% pts with plasma drug levels above threshold—AEs 30% pts	[107]
	NCT02810418	LMB-100 + nab-paclitaxel	Pancreatic cancer	1/2	OR, MTD of short and continous infusion	Completed (2021)—OR 1 pt, OS 89–202 days	[108]
Antibody–drug conjugate	NCT01439152	Anetumab ravtansine	MSLN-positive cancers	1	DLT, pharmacokinetic profile	Completed (2019)—CR 1 (pt); PR (11 pts); SD (66 pts)	[109]
	NCT03126630	Anetumab ravtansine + pembrolizumab	Mesothelioma	1/2	ORR, RP2D	Active, not recruiting	[110]
	NCT02610140	Anetumab ravtansine/vinorelbine	Mesothelioma	2	PFS	Completed (2019)—PFS 4.3 months; OS 9.5 months; ORR 8.4% pts	[111]
	NCT03587311	Anetumab ravtansine + bevacizumab/paclitaxel + bevacizumab	Ovarian, fallopian tube, peritoneal cancers	2	PFS	Active, not recruiting—paclitaxel + bevacizumab showed more benefit	[112]
	NCT02884726	BMS-986148	MSLN-positive cancers	1	AEs, SAEs, grade of AEs, grade of SAEs	Completed (2017)	[113]
	NCT02341625	BMS-986148 + nivolumab	MSLN-positive cancers	1/2a	AEs, SHIFT	Terminated (2020) for business reasons—AEs (125 pts)	[113]
	NCT04175847	RC88	MSLN-positive cancers	1/2a	AEs, MTD, ORR	Recruiting	-
	NCT05508334	RC88	MSLN-positive cancers	1	RP2D	Active, not recruiting	-
	NCT05804526	RC88 + Sintilimab	MSLN-positive cancers	1/2	RP2D	Recruiting	-
	NCT06173037	RC88	Ovarian, fallopian tube, peritoneal cancers	2	ORR	Recruiting	-
	NCT06466187	PF-08052666	MSLN-positive cancers	1	AEs, laboratory abnormalities, dose modifications, DLTs	Recruiting	-
	NCT03507452	BAY2287411	MSLN-positive cancers	1	Incidence of DLTs, AEs, TRAEs, SAEs	Completed (2022)	[114]
Bispecific antibody	NCT05403554	NI-1801/NI-1801 + pembrolizumab/NI-1801 + paclitaxel/paclitaxel	Ovarian, Pancreatic, Non-Small-Cell-Lung and Triple-Negative Breast Cancers	1	DLT, NTD, MTD, PFS, AEs	Recruiting	[115]
	NCT06756035	CT-95	MSLN-positive cancers	1a/1b	MTD, RD, TEAEs	Recruiting	-
	NCT06523803	ZW171	MSLN-positive cancers	1	DLTs, AEs, CRS, neurotoxicity, clinical laboratory abnormalities. ORR	Terminated because of sponsor decision (2025)	-
	NCT06255665	JNJ-79032421	MSLN-positive cancers	1	DLTs, AEs	Completed (2025)	-
	NCT07083323	HY05350	MSLN-positive cancers	1/2	DLT, TEAE, ORR	Not yet recruiting	-
Cell-based therapy	NCT02414269	iCasp9M28z/iCasp9M28z + cyclophosphamide/ cyclophosphamide	Mesothelioma, breast and lung cancer	1/2	severity and number of Aes, clinical benefit rate	Active, not recruiting—SD (8 pts)	[116]
	NCT04562298	LCAR-M23	Ovarian cancer	1	DLTs, TEAEs, MTD, RP2D, CAR-T positive cell concentration	Terminated—Both the sponsors and collaborator are considering terminating the study. (2022)	[117]
	NCT03907852	TC-210 + fludarabine + cyclophosphamide/TC-210 + fludarabine + cyclophosphamide + nivolumab/TC-210 + fludarabine + cyclophosphamide + nivolumab + ipilimumab	MSLN-positive cancers	1/2	ORR, DCR, SD	Active, not recruiting—ORR 20%, DCR 77%, 6-months OS 70%	[11, 118]
	NCT05451849	TC-510 + Fludarabine + Cyclophosphamide	MSLN-positive cancers	1/2	RP2D, ORR, DCR	Active, not recruiting	[119]
	NCT06249256	Fast CAR T cells	MSLN-positive cancers	Early phase 1	DLT	Recruiting	
	NCT05372692	LD013	Ovarian cancer	Not applicable	Objective remission rate	Completed (2023)	-
	NCT03608618	MCY-M11 + Cyclophosphamide	Peritoneal mesothelioma, fallopian tube adenocarcinoma, primary peritoneal carcinoma, adenocarcinoma of the ovary	1	Number and severity of Aes	Terminated because sponsor shift focus (2021)	-
	NCT06562647	SY001	Ovarian cancer	Not applicable	MTD	Recruiting	-
Vaccine	NCT02004262	GVAX Pancreas Vaccine + CRS-207 + cyclophosphamide/CRS-207/Chemotherapy	Pancreatic cancer	2	OS	Completed (2016)—Cy/GVAX + CRS-207 resulted in lower OS than chemotherapy	[120]
	NCT03371381	JNJ-757 + nivolumab/nivolumab	Adenocarcinoma of the lung	1/2	OR	Terminated because of lack of clinical benefits (2018)—PD (83% monotherapy treated pts an 50% combination therapy treated pts)	[121]
	NCT06522919	Autologous Dendritic Cell (DC) Vaccine	Colorectal carcinoma	2	ORR	Recruiting	-

^a^Data were retrieved from clinical trial registry. (https://clinicaltrials.gov/, last visit on 2025/11/19)

^b^*Abbreviations*: *MTD* maximum tolerated dose, *AE* adverse event, *SAEs* serious adverse event, *TEAE* treatment emergent adverse event, *TRAE* treatment-related adverse event, *OR* objective response, *ORR* overall response rate, *OS* overall survival, *DLT* dose-limiting toxicity, *DCR* disease control rate, *SD* stable disease, *PD* progressive disease, *PR* partial response, *RP2D* recommended phase 2 dose, *PFS* progressive free survival, *ADA* anti-drug antibody, *CRS* cytokine release syndrome, *AF* antibody formation, *RD* recommended dose, *SHIFT* Number of Participants With Laboratory Test Toxicity Grade Shifting From Baseline

### Protein-drug conjugates

In addition to immunotoxins, protein-based therapies have included the development of protein-drug conjugates (PDCs) as one of the first strategies for targeting MSLN. PDCs consist of toxic payloads conjugated to mAb that specifically recognizes MSLN [7, 130–132]. In addition to the targeting portion, PDCs incorporate cytotoxic payloads, which typically include microtubule inhibitors, DNA-damaging agents, or topoisomerase inhibitors [133]. The most developed and tested PDCs were based on the use of mAbs, and were therefore named ADCs. However, beyond full-length Abs (~ 150 kDa), drugs have also been conjugated with Ab fragments (antigen binding fragment, Fab ~ 55 kDa; single chain variable fragment, scFv ~ 25 kDa), as well as nanobodies (Nb, ~ 15 kDa) and non-IgG-protein-based scaffolds (8–20 kDa). Due to their small size, these alternative protein scaffolds may enhance penetration into the dense TME, accelerate internalization, or enable the design of multivalent therapeutics while maintaining compact molecular dimensions [104]. In light of these innovations, ADCs are increasingly referred to as protein-drug conjugates (PDCs) encompassing a broader range of emerging therapeutic approaches.

In the context of MSLN-targeting PDCs, six ADCs have progressed to clinical research in patients with MSLN-overexpressing tumors. Among them, Anetumab ravtansine (known also as BAY94-9343) is a fully human IgG1 ab (~ 150 kDa) conjugated with ravtansine, a toxophore able to inhibit microtubules formation [132]. The first in-human Phase I clinical trial of Anetumab ravtansine (AR) started in 2011 and revealed that AR was well-tolerated in MSLN-positive cancer patients (NCT01439152) (Table 1) [109]. Following these promising results, AR was evaluated in multiple clinical trials as monotherapy or in combination with standard therapeutic regimens such as paclitaxel [134]. In some of these studies, the anti-tumor efficacy of AR was compared to chemotherapeutics or immunotherapies. However, results showed limited or no significant improvement over standard treatments, particularly in mesothelioma and OC (NCT03126630, NCT02610140, NCT03587311) (Table 1) [111, 112, 135]. To enhance the efficiency of these ADCs in clinical settings, recent works are focusing on understanding the patient-specific dose depending on a combination of patient-specific variables [136] or on studying interventions (i.e., plasma exchange) that could deplete the impact of soluble MSLN on ADCs efficacy [137]. Another ADC that has reached clinical evaluation is BMS-986148, which consists of a MSLN-targeting monoclonal Ab conjugated to tubulysin. BMS-986148 was tested in patients with MSLN-overexpressing solid tumors (126 enrolled subjects), both as monotherapy and in combination with nivolumab, an immune checkpoint inhibitor. Both treatment strategies were associated with manageable toxicity (13% of patients discontinued due to treatment-related adverse events, AEs) but demonstrated minimal clinical activity with 10 out of 126 patients achieving a partial response (PR) of up to 19.91 months (NCT02341625, NCT02884726) (Table 1) [113]. To improve upon these outcomes, Wittwer and colleagues evaluated the murine equivalent of BMS-986148, called mesoADC, in syngeneic immunocompetent mouse models of colon and breast cancer. In addition to the cytotoxic effect of the tubulysin, mesoADC exhibited immunomodulatory properties. Specifically, it promoted dendritic cell maturation and enhanced tumor-specific T-cell responses while reducing regulatory T-cell (Treg) activity in a tubulysin-dependent manner. Furthermore, mesoADC induced pyroptotic cell death in gasdermin-E-positive tumors when administered as monotherapy [138]. Another anti-MSLN ADC is DMOT4039A, which consists of an IgG1 anti-MSLN mAb h7D9.v3 conjugated with microtubule-disrupting monomethyl auristatin E (MMAE) payload. The maximum tolerated dose (MTD) of DMOT4039A was determined in a Phase I clinical trial involving 71 patients with pancreatic and ovarian cancers. The drug exhibited anti-tumor activity with some patients achieving stable disease (14 subjects) or partial response (5 subjects), and demonstrated a favorable safety profile (7% of patients discontinued due to AEs) following weekly or every-three-week administration (NCT01469793) [139]. In addition, RC88 is currently undergoing clinical evaluation (NCT04175847, NCT05508334, NCT05804526, NCT06173037) (Table 1), following promising therapeutic efficacy observed in a non-human primate model of MSLN-positive tumors [140].

To improve the therapeutic efficacy of ADCs, Cornelison et al*.* attempted to conjugate a monoclonal Ab with a non-tubulysin payload. Indeed, a first-in-class topoisomerase I inhibitor ADC (PF-08052666, also known as SGN-MesoC2 or HBM9033) was developed to exert antitumor activity against MSLN-positive cancers. To enhance the cytotoxicity of PF-08052666 multiple payloads were coupled to the mAb, reaching a drug to antibody ratio of 8. In addition, the tumor specificity of PF-08052666 was ensured through a protease-sensitive linker. PF-08052666 was tested in preclinical models of MSLN-positive tumors, combining MSLN-positive and negative cells. In this context, the novel PF-08052666 outcompeted a canonical tubulysin-based ADC, showing potent antitumor efficacy along with bystander activity. Based on these promising in vivo results, a clinical study is currently evaluating PF-08052666 in patients with mesothelioma, platinum-resistant OC, PDAC, NSCLC, endometrial cancer, and CRC (NCT06466187) (Table 1).

In efforts to improve drug delivery strategies, Yi and colleagues developed an Nb-based system targeting MSLN [102]. This approach utilizes an engineered MSLN-specific Nb (called D3), conjugated to gemcitabine-loaded liposomes (D3-LNP-GEM) combining enhanced tissue penetration via liposomes with active targeting through the Nb. D3-LNP-GEM showed promising anti-cancer efficacy, reducing tumor volume by approximately 70% in a pancreatic cancer mouse model, and showing greater accumulation within TME compared to LPN-GEM alone. Moreover, D3-treated cells showed reduced expression of two EMT promoters (TWIST1 and FN1), which are known to correlate with the MSLN expression. Their downregulation suggests a novel mechanism of action of MSLN targeting agents, potentially involving EMT modulation and suppression of cancer progression [102]. Towards the goal of developing new therapeutic strategies, a small PDC was recently engineered using a non-IgG1 scaffold based on Designed Ankyrin Repeat Proteins (DARPins) [141]. The resulting anti-MSLN DARPin, called M7A-DC, was conjugated to MMAE, a tubulin inhibitor, and tested in a preclinical pancreatic cancer model. M7A-DC showed strong tumor penetration and potent anti-tumor activity, even in low-MSLN-expressing tumors and when combined with immune checkpoint inhibitors. Notably, its therapeutic efficacy was independent of sMSLN, which typically acts as a decoy and reduces ADC efficiency [141]. These findings underscore the translational potential of non-antibody-based therapeutic platforms in targeting MSLN-expressing malignancies.

A subset of the toxic payload conjugated to Abs or proteins includes radionuclides, which enable MSLN-directed molecular imaging and radio-targeted therapy [142]. This strategy supports patient stratification and the administration of personalized treatments based on individual MSLN expression levels. In this context, a thorium-227 conjugated Ab, BAY2287411, was developed and evaluated in patient-derived xenograft models of various MSLN-overexpressing malignancies, including breast, colorectal, lung, ovarian, and pancreatic cancers. The preclinical results were encouraging, supporting progression to clinical investigation [114, 143]. A first-in-human clinical trial of BAY2287411 involving 36 patients affected by mesothelioma, ovarian, and pancreatic cancers has been completed, with results available  concerning safety, tolerability, and anti-tumor activity (NCT03507452) (Table 1). Pursuing a similar objective, the well-known chimeric therapeutic mAb Amatuximab [144] was conjugated with Zirconium-89 for non-invasive diagnostic imaging and with Lutetium-177 for therapeutic purposes. These radio-labeled Amatuximab complexes were tested in a pancreatic cancer xenograft model, showing MSLN-specific accumulation and a significant reduction in tumor mass, accompanied by overall toxicity [145]. In the field of molecular imaging, reducing the size of the radiotracer could enhance tumor penetration and decrease background signal due to the rapid clearance of smaller molecules. To this end, Benloucif and colleagues recently engineered an Nb (Nb S1, ~ 15 kDa) targeting MSLN. Nb S1 was conjugated with Gallium-68, and its biodistribution was assessed both in vivo and ex vivo, revealing high tumor uptake and a low signal-to-background ratio [146]. Furthermore, our research group is engineering a novel MSLN-directed radiotracer based on the fibronectin type III scaffold (Fn3 5.3.2) for theranostic application in MSLN-positive cancers. Fn3 5.3.2 has shown rapid internalization, high specificity and affinity for MSLN-positive mesothelioma cells, as well as capability of bioconjugations to radiometal chelators, paving the way for the development of MSLN-targeting radio-Fn3 moieties [147]. Overall, these studies highlight how PDC design is evolving toward the development of therapeutic molecules capable of effectively reaching their target while maintaining functionality within the complex tumor microenvironment.

### Bispecific therapeutic agents

In the last decade, Abs have been engineered to create molecules with multiple mechanisms of action, including the ability to target different epitopes of the same antigen or distinct antigens simultaneously. This strategy enables the combination of multiple targeting activities within a single therapeutic agent, thereby enhancing anti-cancer potential [148]. These engineered molecules are commonly referred to as bispecific antibodies (bsAbs). In the context of MSLN-targeted therapies, bsAbs have been engineered to simultaneously bind a tumor-associated antigen, such as MSLN, and a co-stimulatory receptor expressed on T-cells (*e.g.,* CD3, CD20, CD47, CD137) [149]. This dual-targeting approach facilitates the recruitment and activation of cytotoxic immune cells at the site of MSLN-expressing tumors, thereby enhancing immune cells proliferation and cytotoxic activity [150, 151]. Following this rationale, Ye and colleagues developed a bispecific antibody targeting MSLN and CD40 (referred to as ABBV-428) and evaluated its efficacy in preclinical models [152]. Targeting CD40 with a bsAbs can activate the CD40-dependent signaling pathways that support the recruitment and expansion of tumor-specific T-cells [153]. Indeed, ABBV-428 efficiently induces T-cell activation and reduced tumor growth in a MSLN-dependent manner.

This promising therapeutic candidate was evaluated in a phase I clinical trial including 59 patients with MSLN-positive advanced solid tumors, with mesothelioma and OC patients included in the dose expansion phase (NCT02955251) (Table 1). In this context, Luke et al. reported that ABBV-428 was well tolerated but showed limited anti-tumor activity (47% subjects had stable disease as best response); therefore, further clinical evaluations with higher doses of ABBV-428 are planned [154]. Pursuing a similar goal, Liu and colleagues recently engineered a bsAb targeting MSLN and CD3 (MSLN490) that enhanced the recruitment of CD3-expressing T-cells [11]. In vitro assays showed strong therapeutic activity of MSLN490. In addition, a significant reduction of MSLN-positive tumor masses was observed in mice treated with a combination of MSLN490 and standard chemotherapeutics (*i.e.,* paclitaxel), suggesting that this strategy is promising even for solid tumors with low immune cell infiltration [11].

Another dual-targeting strategy involves targeting CD47, a molecule that inhibits immune-mediated clearance of both healthy and tumor cells. Given CD47’s role in immune evasion, a MSLN-targeting approach was developed to block CD47-dependent escape mechanisms in cancer cells [115]. The bsAb NI-1801 was engineered to simultaneously recognize a membrane-proximal epitope on MSLN and CD47. In vivo studies using hepatocarcinoma xenograft models treated with NI-1801 showed effective tumor control, with significantly reduced tumor mass compared to mice receiving a monospecific MSLN-targeting Ab. Interestingly, the study revealed that targeting specific MSLN epitopes can influence not only binding affinity but also immune cells recruitment and antibody-dependent cellular phagocytosis [115]. Based on these findings, NI-1801 is currently being evaluated in a Phase I clinical trial (NCT05403554) (Table 1), which is actively recruiting patients with MSLN-positive solid malignancies. In this context, NI-1801 is evaluated as a monotherapy and in combination with anti-PD1 agent pembrolizumab or the standard chemotherapeutic paclitaxel. As most bs Ab-based therapies remain in early-phase clinical development, additional strategies are under investigation to improve target specificity and minimize adverse side effects such as on-target/off-tumor toxicity and cytokine release syndrome [155]. To enhance specificity to membrane-bound MSLN, bsAbs can be designed to target the membrane-proximal region of MSLN (MSLN_MPR_), which remains intact following proteolytic cleavage. This strategy has shown superior efficacy compared to bsAbs directed against the membrane-distal region of MSLN (MSLN_MDR_) that is often shed into the bloodstream or present in non-tumor tissues, potentially leading to on-target/off-tumor toxicity (NCT06756035, NCT06523803, NCT06255665) (Table 1). For example, Hatterer and colleagues demonstrated in vivo that a MSLN_MPR_ × CD47 Abs has higher efficacy in recruiting T-cell but also natural killer (NK) cells with respect to MSLN_MDR_ × CD47 or to the bench standard Amatuximab [115]. Similarly, Chakraborty et al. engineered an MSLN_MPR_ × CD3 bispecific antibody that showed potent tumoricidal activity and minimal interference from sMSLN [156]. Enhancing targeting precision in immune-cell engagers is critical to promote immune cell proliferation within the TME in solid tumors, which is often enriched with immunosuppressive factors [155]. To address this challenge, a tri-specific antibody targeting MSLN, CD3, and PD-L1 was developed, and it is currently evaluated in a Phase I/II clinical trial (NCT07083323) (Table 1).

### MSLN-directed cell-based therapies

Improving immune cell co-stimulation in tumors is also one of the motivations behind the development of CAR-based therapies [157]. Indeed, the latest generations of CAR are equipped with intracellular domains that trigger signaling not only for the recruitment but also for the expansion and proliferation of immune cells [157]. Enhancing CAR-T cells activation is of primary importance for the treatment of solid tumors, since the immunosuppressive TME tends to exhaust CAR-bearing T cells, decreasing their therapeutic efficacy [12, 158]. In this regard, in a clinical trial whose results are publicly available, Adusumilli et al. tested a CAR-T cell system directed against MSLN in patients with mesothelioma [116]. In combination with cell-based therapy, a programmed cell death protein 1 (PD-1) inhibitor, pembrolizumab, was administered to 23 patients with mesothelioma (NCT02414269). PD-1 is known to reduce immune response upon binding with its ligands (PD-L1 or PD-L2), thus repressing T-cell response in the tumor environment [159]. Using a combination of therapeutics to target PD-1 can reduce PD-1/ligands interactions, resulting in higher T-cell proliferation and anti-tumor effect of CAR-T cells [160, 161]. Adusumilli et al*.* showed an increased clonal expansion of CAR-T cells thanks to the combination of cell therapy and pembrolizumab in 39% of treated patients. Notably, CAR-T cells persisted in the bloodstream for ≥ 100 days following pembrolizumab administration, demonstrating long-lasting proliferation. Thanks to these promising results, a new clinical study is underway to evaluate a fixed-dose regimen of anti-MSLN CAR-T cells followed by pembrolizumab after four weeks (NCT02414269) (Table 1).

To counteract immune suppression mediated by TGF-β within the TME, Li and colleagues engineered CAR T-cells co-expressing an anti-MSLN single-domain antibody and a dominant-negative TGF-β receptor type II (dnTGFβRII) [117]. This decoy receptor effectively blocks TGF-β signaling and the consequent immune suppression. The resulting MSLN × TGF-β CAR-T cell system showed potent antitumor efficacy in both in vitro and in vivo models of OC, with enhanced T-cell persistence in the TME [117]. Building on these promising findings, a clinical trial was conducted to evaluate the safety and pharmacokinetics of this CAR-T cell therapy in patients with epithelial ovarian cancer, with results currently pending (NCT04562298) (Table 1).

Recently, novel strategies based on chimeric T-cells have been developed to enhance T-cells persistence within the TME while minimizing therapy-related side effects. Among these, Ding and colleagues proposed a T-cell receptor fusion construct (TRuCT®) generated by fusing an anti-MSLN single-chain variable fragment (scFv) to a T-cell receptor [118]. Unlike CAR-T cells, the antigen-binding domain is linked to a subunit of the TCR complex, and it can participate in the interplay of the six co-stimulatory subunits of the TCR [162]. Thanks to enhanced signaling, the anti-tumor response mediated by TRuCT® cells was observed to be more efficient than that of conventional CAR-T cells. In the anti-MSLN TRuCT cells, the antigen-binding domain is linked to the CD3_ε,_ showing less exhaustion of T-cells and lower cytokine release [118]. Hassan and colleagues evaluated anti-MSLN TRuCT cell therapy in a phase I/II clinical trial (NCT03907852), reporting safety and feasibility of this therapeutic approach in patients with MSLN-positive refractory tumors [11] (Table 1).

To further enhance the efficacy of the anti-MSLN TRuCT, McCarthy and colleagues added a PD1-CD28 chimeric switch receptor (CRS) to the construct [119]. This receptor converts the inhibitory signal typically mediated by PD-1/PD-L1 interaction into a stimulatory signal via the CD28 domain [163, 164]. This more comprehensive approach, which integrates multiple co-stimulatory subunits, resulted in enhanced T-cell persistence compared to the TRuCT cells not bearing the CRS [119]. This evidence highlights the importance of considering immune suppression in the TME when designing and testing novel drugs, especially in solid tumors. Furthermore, the anti-tumor efficacy of CD28-PD1 CRS expressed in TRuCT cells was validated in mice bearing MSLN-positive tumors, and this strategy is currently being investigated in a phase I/II clinical trial (NCT05451849) (Table 1). Working to prolong persistence of CAR-based therapies in the TME, Sun and colleagues developed a non-viral JL-Lightning-CAR-T cell-based therapy (known as BZT2312), which combines MSLN targeting domains with the secretion of anti-PD-L1 Nb. The Nb secretion was conceived to neutralize immunosuppression in MSLN-positive cancers. BZT2312 is undergoing a pilot phase I clinical study, enrolling patients with MSLN-positive pleural mesothelioma (NCT06249256) (Table 1). Interestingly, a clinical study conducted in patients with MSLN-positive drug-resistant, relapsed ovarian cancer showed differences in metabolic profiles between patients with high or low response to an anti-MSLN CAR-T therapy (known as LD013) (NCT05372692) (Table 1). Among the metabolites analyzed, glutamine levels were found to be reduced in the non-responder group. Following these results, Chen and colleagues pre-treated LD013 with glutamine to check for potential metabolic rewiring in vitro and in preclinical models [165]. Pre-treated LD013 demonstrated higher therapeutic potential, persistence, and proliferation compared with non-treated LD013 in xenograft models of OC, owing to glutamine-dependent increases in membrane stability and immune synapse formation [165]. These findings suggest that metabolic regulation can reveal key factors underlying poor therapeutic response, paving the way toward combined and more effective strategies.

For their innate anti-tumor role and the high infiltration in TME, macrophages were proposed as candidate immune cells to be engineered to express CARs for therapeutic approaches [166]. The first-in-human clinical trial investigating MSLN-targeting CAR-macrophages (CAR-M) was proposed by Annunziata and colleagues, who studied peripheral blood mononuclear cell (PBMC)-derived CARs (MCY-M11), including macrophages, in patients with OC and mesothelioma (NCT03608618). This preliminary evaluation showed the feasibility of rapidly produced MCY-M11, with low cytotoxicity following administration in 11 patients (no dose-limiting toxicity or high-grade CRS detected). After one cycle of treatment, patients reported stable disease (NCT03608618) (Table 1). Li and colleagues proposed another MSLN-targeting CAR-M, generated by adenoviral transfection of patient-derived macrophages (CAR-pMAC, namely SY001) [167]. SY001 induced near-complete regression of MSLN-positive tumors in xenograft models after intraperitoneal administration, demonstrating improved anti-tumor efficacy compared to intravenous infusion [167]. In addition, the safety profile of SY001 was preliminarly evaluated in two OC patients, revealing no high-grade AEs while achieving disease stabilization. Further clinical investigations are ongoing to enhance SY001 efficacy through intraperitoneal injections in combination with anti-PD1 therapeutics (NCT06562647) (Table 1).

Ongoing preclinical studies are exploring new paths to overcome the limitations encountered by engineered immune cells in solid tumors. Indeed, the latest generation of CARs is engineered with multiple co-stimulatory domains to enhance T cell proliferation and cytotoxicity. Consequently, if CARs encounter sMSLN or do not reach the target in the dense stroma of solid tumors, the enhanced cytotoxic effect can be directed to healthy tissues, causing side effects (cytokine release syndrome) [168] or rapid exhaustion of CARs immune cells. To improve delivery precision within tumor site, Zheng et al. generated an inhalable exosomes-based system of anti-MSLN CAR-T cells and paclitaxel. In a preclinical study of lung adenocarcinoma, this inhalable therapy demonstrated potent anti-tumor efficacy, showing that combining multiple therapeutics can reduce CAR-T cells or paclitaxel accumulation in liver and spleen, overcoming systemic cytotoxicity derived from canonical delivery systems (*e.g.* injection) [169]. Trying to bypass the sMSLN-related tumor resistance to CAR-T cells, Liu and colleagues built an anti-MSLN CAR-T (15B6 CAR-T) with an Ab recognizing an MSLN epitope close to the membrane that is not affected by MSLN shedding. H15B6 CAR-T showed a potent anti-tumor effect in a pancreatic cancer mouse model. In contrast, the activity of CAR-T targeting a membrane distal region (SS1 CAR-T) showed a reduced accumulation and anti-tumor effect, because the SS1 CAR-T cells were also recruited and inactivated by sMSLN [170]. In another study, the MSLN membrane-proximal region was targeted by hYP218 scFv, which served as the recognition domain in a CAR-NK cell system. CAR-NK cell-based therapies may offer enhanced immune cell proliferation and reduced exhaustion compared to conventional CAR-T approaches. Interestingly, these CAR-NK (namely MSLN.CAR-IL-15 GR1.1-iNK cells) were derived from induced pluripotent stem cells (iPSCs) and engineered to express an IL-15 module for enhanced proliferation combined with the anti-MSLN hYP218 scFv. After effective in vitro depletion of the tumor, MSLN.CAR-IL-15 GR1.1-iNK cells were evaluated in a PM mouse model, resulting in potent anti-tumor activity guaranteed by enhanced infiltration and proliferation in tumor masses [171]. In another study, iPSCs were used to generate CAR-M (namely CAR-iMACs), which were further engineered to enhance their anti-tumor activity. To this end, Wang and colleagues sought to increase the pro-inflammatory activity of CAR-iMACs by CRISPR-mediated silencing of genes that were identified as potential pro-inflammatory markers (*e.g., ACOD1*) through metabolic screening. In a preclinical mouse model of OC, *ACOD1*-knockout CAR-iMAC exhibited enhanced pro-inflammatory activity and improved anti-tumor efficacy when combined with immune checkpoint inhibitors [172]**.**

### Vaccine-based therapies

MSLN-directed tumor vaccines are a growing field aimed at eliciting robust anti-cancer immune responses. Due to their typically low immunogenicity, vaccines are often combined with other therapeutic modalities to enhance efficacy [173]. One such approach involves the use of attenuated *Listeria monocytogenes* to generate an MSLN-expressing vaccine (CRS-207), which was tested in patients with MSLN-positive tumors either as monotherapy or in combination with GVAX. In Phase IIb clinical trials, CRS-207 (58 subjects) was compared with standard chemotherapeutics (43 patients) but did not achieve the initial aim of improving overall survival over chemotherapy (NCT02004262) [120] (Table 1). Another attenuated *Listeria monocytogenes* vaccine (JNJ-757) showed limited efficacy and clinical benefit in two Phase I studies involving patients with MSLN-positive advanced lung adenocarcinoma, whether administered alone (n = 18) (NCT03371381) or in combination with nivolumab (N = 12) (NCT02592967). As a result, further development of this combined therapy was discontinued [121] (Table 1).

Beyond attenuated bacterial vaccines, dendritic-cells (DCs)-based vaccines represent another promising avenue. A novel Phase II clinical trial is currently recruiting to evaluate a combination therapy that includes a DC-based vaccine for CRC (NCT06522919). To address the limited availability of DCs and improve reproducibility in vaccine production, Tominaga and colleagues developed a vaccine using MSLN-targeting iPSC-derived DCs (iPSCDCs), which successfully induced cytotoxic T-lymphocytes (CTLs) in an MSLN-dependent manner [174] (Table 1).

Another innovative approach involves the use of personalized neoantigens as immune activators. After identifying a correlation between MSLN expression and poor cytotoxic T cell recruitment in the pancreatic cancer microenvironment, *Cai *et al*.* demonstrated in a mouse model that targeting MSLN with a neoantigen peptide vaccine enhanced immune cell infiltration into the TME [103]. This study highlights the potential of MSLN-targeted vaccines to modulate the immunosuppressive TME in solid tumors.

### Current challenges in MSLN-targeted therapy

Redirecting toxic payloads or immune cells to MSLN for therapeutic purposes is a growing field of research, which faces the inherent challenges of targeting solid tumors. Solid tumors are characterized by high variability in architecture and development. Mechanistically, the complex stroma of solid tumors is enriched in molecules, vasculature, and immune cells, whose interplay can affect the fate of therapeutics, acting as both chemical and physical barriers. Since the adoption of earlier generations of therapeutics such as mAbs (*i.e.,* Amatuximab), early ADCs, or immunotoxins, immunosuppression and dense ECM have been identified as limiting factors for the successful outcome of MSLN-directed therapeutics. Thanks to the efforts of researchers, multiple variables of the TME have been decoded, and novel anti-tumor pharmaceuticals are exploiting this knowledge to target solid tumors. In Section “Mesothelin targeted therapies”, we described combination therapies, bispecific antibodies, and cell-based approaches that have evolved to overcome immune checkpoint inhibitors or to recruit immune cells against tumors [115, 117–119, 171, 175]. Furthermore, protein-based therapeutics have been developed to generate small targeting agents (~ 10–20 kDa) with enhanced tumor penetration and faster internalization into cancer cells [102, 141, 146, 147].

As described in Section “Mesothelin in tumor pathology and dynamics” of this review, MSLN plays a role in tumor progression and metastasis, and can be found either anchored to the membrane or in its soluble form (Fig. 1). This dual behavior represents one of the current challenges for MSLN-targeted therapeutics. Indeed, sMSLN acts as a decoy for MSLN-targeted therapeutics, reducing therapeutic efficacy or causing off-tumor toxicity in patients. As described in Section “Mesothelin targeted therapies”, multiple studies have attempted to overcome the sMSLN issue by targeting the portion of MSLN that is not cleaved or by focusing on proteases-sensitive regions, trying to reduce MSLN shedding [141, 156, 169, 170]. These strategies resulted in efficient and specific antitumor activity, unaffected by sMSLN, when applied in antibody- or cell-based therapies.

A critical factor to consider in clinical studies is the low level of MSLN in healthy tissues. Basal expression of MSLN can become a major issue, as therapeutics may be redirected to healthy tissues, causing off-target/off-tumor toxicity [176]. This clinical outcome necessitates lowering therapy doses and influences the choice of administration routes. To avoid off-tumor effects in patients, several strategies have been applied, including dual targeting approaches and the implementation of cell-based therapies with highly specific co-stimulatory domains. Nevertheless, off-tumor toxicity remains a challenge and a complex event to elucidate, requiring improved preclinical investigations to mitigate its impact with MSLN-positive tumors.

Overall, MSLN remains a compelling therapeutic target in solid tumors, particularly when combined with strategies that counteract immunosuppression within the TME. Anti-MSLN therapies necessitate continuous implementation to face the ever-new unfulfilled goals in current clinical investigations. Future studies will clarify their clinical impact and optimize their integration into multi-modal treatment regimens.

## Conclusions

In this review, we provided an overview of MSLN biology and its role in each aspect of cancer development, from the establishment of an oncogenic TME to the enhancement of the metastatic features of cancer cells. We also discussed current MSLN-based therapeutic approaches, including their advantages and limitations.

Studies conducted to elucidate the expression pattern of the MSLN gene, especially in a malignant context, have identified peculiar regulatory elements, including a specific methylation pattern observed in PDAC, and miRNA-mediated silencing. Additionally, a specific sequence known as CanScript has been reported to strongly enhance MSLN expression in several malignancies by interacting with a range of cancer-specific transcription factors.

In recent years, a growing body of evidence has contributed to shedding light on the mechanisms through which MSLN promotes cancer progression. MSLN has been reported to facilitate both cell–cell and extracellular matrix adhesion by interacting with CA125, thereby promoting peritoneal dissemination in MSLN-overexpressing cancers. Additionally, MSLN has been associated with specific features of the peritoneal basal membrane, including decreased collagen density and increased fenestrations, thereby enhancing tissue permeability to cancer cells.

Another route through which MSLN contributes to the metastatic spread is by affecting the extracellular matrix degradation. In this context, MSLN has been reported to act mostly by promoting MMP-7 expression through both CA125-dependent and -independent mechanisms that activate the MAPK axis. Additionally, MSLN may promote EMT, offering an alternative route for enhancing metastasis, invasion, and cell migration, an area that warrants further investigation.

We also discussed the association between MSLN and specific TME characteristics, as well as its role in reshaping the TME towards immune-suppressive features. The most recent data on this topic have shown that MSLN can also drive macrophage polarization towards an immunosuppressive phenotype characterized by the secretion of VEGFA, ARG1, and S100A9, thereby stimulating cancer cell growth and weakening the immune response.

Finally, we reviewed the most recent MSLN-targeting strategies, including bispecific antibodies, CAR-T cells, and anti-MSLN TRuC-T cells. Among these, bispecific antibodies represent the most clinically advanced approach, with several candidates currently undergoing Phase I/II evaluation. However, none of the available MSLN-targeting therapies have been approved for clinical use yet, highlighting the need for more effective strategies that overcome TME-mediated immune suppression, enhance specificity, and minimize off-target effects.

Taken together, these data highlight the multiple roles of MSLN in cancer biology and its promise as a therapeutic target. Further research should aim at deepening the knowledge of the regulatory networks driving MSLN expression, clarifying its interaction with the TME, and refining therapeutic approaches to fully exploit the potential of MSLN-targeting therapies.

## Data Availability

All data supporting the findings of this study are included within the paper.
